# Quantification of Endogenous Steroids and Hormonal Contraceptives in Human Plasma via Surrogate Calibration and UHPLC-MS/MS

**DOI:** 10.1021/acs.analchem.5c01912

**Published:** 2025-06-20

**Authors:** Min Su, Bernhard Drotleff, Tamara Janker, Zoé Bürger, Ann-Christin S. Kimmig, Birgit Derntl, Michael Lämmerhofer

**Affiliations:** † Institute of Pharmaceutical Sciences, Pharmaceutical (Bio-)Analysis, University of Tuebingen, 72076 Tuebingen, Germany; ‡ Metabolomics Core Facility, EMBL Heidelberg, 69117 Heidelberg, Germany; § Department of Psychiatry and Psychotherapy, Tübingen Center for Mental Health (TüCMH), University of Tübingen, 72076 Tübingen, Germany; ∥ Department of Women’s and Children’s Health, Science for Life Laboratory, Uppsala University, 75185 Uppsala, Sweden; ⊥ LEAD Research School and Graduate Network, University of Tuebingen, 72074 Tuebingen, Germany

## Abstract

Quantifying endogenous and exogenous steroids at low concentrations in biological matrices remains a major analytical challenge. Immunoassay-based diagnostics are limited by cross-reactivity, particularly at low levels, prompting a shift toward (ultra)­high-performance liquid chromatography–tandem mass spectrometry ((U)­HPLC-MS/MS) for clinical applications. A key limitation for endogenous hormone quantification is the absence of a true blank matrix for external calibration. To address this, we developed a surrogate calibration method employing 1,2-dimethylimidazole-5-sulfonyl chloride (DMIS) derivatization for estrogens, enabling sensitive and selective quantification alongside nonderivatized steroids. Stable isotope-labeled surrogate calibrants and internal standards were used to achieve matrix-matched quantification within a clinically relevant range. Parallelism between analytes and surrogate calibrants was systematically verified in plasma across multiple calibration levels. The method was further optimized through the use of narrow-bore UHPLC columns and refined chromatographic conditions to enhance sensitivity and resolution for a broad analyte panel. Combined with efficient protein precipitation and 96-well plate-based solid-phase extraction, the developed assay achieves pg/mL-level quantification in human plasma with high precision and accuracy. This integrated approach uniquely combines surrogate calibration for endogenous steroids with external calibration for exogenous contraceptives, including sensitive DMIS-based derivatization for estrogens, enabling comprehensive hormonal profiling in a single run. Beyond its analytical scope, the method outlines a structured validation strategy, which is aligned with regulatory principles, and may therefore serve as a practical reference for future LC–MS/MS assays employing surrogate calibration.

## Graphical abstract

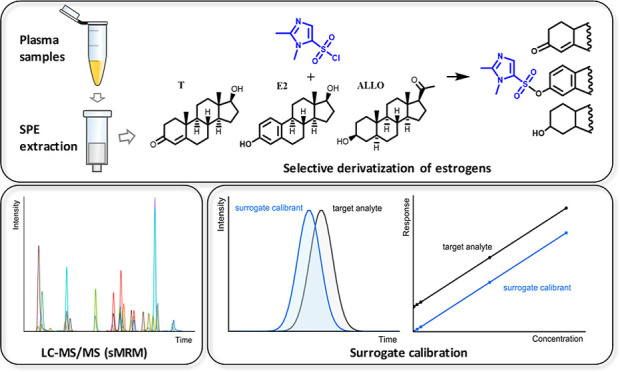

Steroid hormones are essential regulators of various physiological processes, such as water-salt balance, stress response, immune response, sexual differentiation, and reproduction.^1^ Both naturally occurring and synthetic steroids serve as potent signaling molecules and are routinely assessed in diagnostics and clinical studies.^2–4^ Despite the widespread use of immunoassays for steroid analysis, concerns have been raised regarding their reliability due to well-documented limitations, such as limited specificity, cross-reactivity, matrix effects, and the potential for false underestimation caused by the Hook effect.^5,6^ These limitations have led to increasing scrutiny and debate regarding the accuracy and reliability of steroid immunoassays in clinical applications.^7,8^ To address these challenges and achieve accurate and sensitive steroid analysis, liquid chromatography coupled with mass spectrometry (LC–MS/MS) has emerged as a powerful alternative.

LC–MS/MS is a well-established technique for steroid analysis, valued for its fast analysis time, high specificity, minimal sample volume, and broad analyte coverage within a single injection. Steroid separation is typically performed using reversed-phase (RP) columns with C8, C18, or phenyl-modified silica stationary phases.^9–12^ To enhance separation efficiency, improve sensitivity, and shorten analysis time, ultrahigh-performance liquid chromatography (UHPLC) with sub-2 μm particle columns and core–shell particle technology have been increasingly utilized.^13,14^ An additional option to enhance sensitivity for challenging applications is the usage of columns with a narrow internal diameter (ID), e.g. 1.0 mm ID columns.^15^ Besides increased sensitivity due to higher analyte concentration during detection and improved ionization efficiency, 1.0 mm ID columns also drastically reduce solvent consumption by requiring lower flow rates. However, they present challenges such as limited loading capacity and susceptibility to extra-column band broadening, requiring careful system optimization to fully leverage their benefits.

Triple-quadrupole mass spectrometers, which are classically operated in multiple-reaction monitoring (MRM) mode to simultaneously record selective precursor-to-fragment transitions, have been extensively utilized in the quantitative analysis of steroids by LC–MS/MS.^11,16,17^ Furthermore, the application of scheduled MRM (sMRM) mode allows for the detection of a broader range of analytes with maximum dwell time in a single analytical run while maintaining at least 12 data points per peak of interest, ensuring reliable quantification without compromising sensitivity.

Absolute quantification of endogenous compounds, such as steroids in plasma, using LC–MS/MS remains challenging due to the absence of truly analyte-free biological matrices. Several strategies have been adopted to address this issue.^18–20^ Surrogate calibration was selected for this study as it enables precise and accurate quantification in true matrix with distinct benefits compared to alternatives like standard addition, background subtraction, and surrogate matrix approaches.^12,21–25^ Background subtraction is prone to inaccuracies, especially when quantifying concentrations below background level, while standard addition is time-consuming, involves extrapolation (with susceptibility to large variance caused by outliers), and requires larger sample volumes. In contrast, surrogate calibration is the most robust to control matrix effects and the only approach that allows reliable determination of LODs, LOQs and linear ranges of target analytes in the matrix. By spiking stable-isotope-labeled analogue (SIL) into the true matrix, the surrogate calibrants (often termed surrogate analytes in the literature) closely mimic the behavior of the target analytes. After initial response matching of the SIL to target analytes, and verification of parallelism, the concentration of the endogenous analyte is determined using the regression equation derived from the surrogate SIL calibration curve.^26,27^

The most commonly used extraction techniques for steroid analysis include protein precipitation (PP),^28,29^ liquid–liquid extraction (LLE),^30–32^ 96-well plate supported liquid extraction (SLE),^33^ and solid-phase extraction (SPE).^12,34,35^ Also, a one-step sample preparation combining PP with phospholipid removal by off-line SPE (with zirconia metal oxide-coated silica) achieved adequate performance for steroid analysis in clinical laboratories.^36^

Precolumn derivatization is another widely used technique to improve the sensitivity,^37–39^ particularly for quantifying estrogens in e.g. plasma from female administering hormonal contraceptives, where endogenous estrogen levels are markedly suppressed. As reported, derivatization with reagents such as dansyl chloride,^10,40,41^ hydroxylamine,^42^ picolinoyl chloride hydrochloride,^43^ or *p*-toluenesulfonylhydrazide^44^ introduces additional functional groups to either phenol or keto moieties of estrogens, enhancing ionization efficiency and altering fragmentation patterns and chromatographic retention.

In the present study, we developed an LC–MS/MS-based method for the analysis of endogenous and exogenous hormones in plasma. 1,2-Dimethylimidazole-5-sulfonyl chloride (DMIS)^45,46^ was used for the selective derivatization of estrogens. It showed improved sensitivity and specificity due to its estrogen-specific fragmentation. To ensure accurate and precise quantification, ^13^C-labeled and deuterated SIL analogues were used as surrogate calibrants and internal standards, respectively. In order to maximize sensitivity for the challenging target analyte class, we combined PP and SPE for analyte purification, followed by concentration through evaporation and low-volume reconstitution. Furthermore, the method incorporated the usage of 1.0 mm ID columns and selective DMIS derivatization of estrogens, allowing for pg/mL-level quantification of 12 endogenous and 5 exogenous hormones by simultaneously detecting derivatized estrogens and nonderivatized steroids. This work demonstrates that surrogate calibration can be effectively applied to a large panel of targets simultaneously and is the first to showcase its feasibility in combination with derivatization. Considering the multiple challenges in steroid quantification of endogenous and exogeneous steroids, optimization of a number of analytical details (SPE, derivatization, narrow-bore column, surrogate calibration) has led to an optimized robust steroid assay.

Ultimately, in the absence of formal regulatory guidance on surrogate calibrant-based quantification, this study established a structured validation framework aligned with FDA bioanalytical principles. Key elements included verification of parallelism between surrogate calibrants and their respective analytes, response factor adjustment through fine-tuning of CE/DP settings and/or calibrant concentration, and matrix effect control using appropriate internal standards. The method’s robustness was confirmed through accurate quantification of certified commercial quality controls, supporting its reliability for both clinical and research applications.

## Experimental Section

### Sample Preparation

Blood samples were collected from female participants following a psychosocial stress induction with the Maastricht Acute Stress Task (MAST).^47^ Subjects providing blood samples gave written informed consent to the study that conformed to the Declaration of Helsinki as revised in 2013 and was approved by the local Ethics Committee of the Medical Faculty Tübingen (ethics approval number 067/2020).

After centrifugation at 4400 rpm for 15 min, the surface plasma samples were aliquoted into 500 μL portions in 2 mL polypropylene Eppendorf tubes. Those aliquots were stored at −80 °C and thawed on ice for 4 h prior to further processing. Protein precipitation and analyte extraction were obtained by addition of 1 mL of a MeOH/50 mg/mL ZnSO_4_ in H_2_O (80/20, v/v) mixture^48^ that contained the following labeled analytes as internal standards: ChAc-*d*_6_ (0.2 ng/mL), cortisone-*d*_8_ (16 ng/mL), dienogest-*d*_8_ (0.8 ng/mL), E1-^13^C_6_ (0.05 ng/mL), EE2-*d*_4_ (0.05 ng/mL), LNG-*d*_6_ (0.1 ng/mL), P-*d*_9_ (0.1 ng/mL), 17OHP-*d*_8_ (0.1 ng/mL). After vortexing for 15 s and equilibration on ice for 15 min, the samples were centrifuged for 10 min at 15,000 ×*g* and 4 °C with a 5415R microcentrifuge (Eppendorf, Hamburg, Germany). The supernatants were loaded onto a dry Oasis PRiME HLB SPE 96-well plate cartridge (1 cc/30 mg, Waters, Milford, MA, USA). SPE was carried out by applying positive pressure (N_2_; 3–6 psi for loading, washing, and elution, 25 psi for drying) with a PPM-96 (positive pressure manifold 96 processor, Agilent Technologies, Waldbronn, Germany). Subsequently, the loaded cartridges were washed with 1 mL of ice-cold 50% MeOH in H_2_O (v/v). After drying for 5 min, analytes were eluted with 2 × 300 μL MeOH (ambient temperature) into an ACQUITY UPLC 700 μL round 96-well sample collection plate with conical bottom shape (Waters). The eluates were dried under N_2_ for 8 h using an EZ-2 evaporator (Genevac, Ipswich, UK). Derivatization with DMIS^43^ was carried out by addition of 35 μL sodium carbonate-bicarbonate buffer (50 mM, pH 10.5) and 15 μL DMIS (1 mg/mL) in acetone to each well. Followed by the immediate closing of the 96-well plate with a pierceable Captiva 96-well collection plate cover mat (Agilent Technologies) the derivatization mixtures were incubated in a Thriller Thermoshaker Incubator (VWR, Radnor, PA, USA) at 25 °C and 1400 rpm for 15 min. Afterward, the 96-well plate was centrifuged for 10 min at 1500 ×*g* with a GS-6R centrifuge (Beckman Coulter, Brea, CA, USA), subsequently it was placed into the thermostated autosampler (4 °C) and samples were analyzed as soon as possible (start of the sequence maximum 2 h after preparation).

### LC–MS Method

The chromatographic instrumentation consisted of a 1290 Infinity II LC and Multisampler system (G7120A and G7167B, Agilent Technologies). Separation was performed on a Kinetex XB-C18 column (100 mm × 1.0 mm, 2.6 μm, 100 Å pore size) with a KrudKatcher Ultra in-line filter for column protection (both Phenomenex, Torrance, CA, USA). The mobile phase was delivered at a flow of 0.1 mL/min. A column temperature of 30 °C was maintained in an external column oven (MicroLC 200 oven, Sciex, Concord, Ontario, Canada), which was installed in close proximity to the ion source inlet to reduce extra-column volume. Mass spectrometric detection was performed on a QTRAP 4500 mass spectrometer with a Turbo V electrospray ionization (ESI) source (Sciex). Further details about LC–MS settings are provided in the Supporting Information and summarized in Table S1. The analytical system was controlled by the Analyst 1.7.1 software (including Analyst Device Driver 1.3, Sciex).

### Data Analysis and Quantification

The concentrations of each calibration level and quality control (QC) sample are listed in Table S2. Calibration curves were determined using weighted least-squares linear regression of seven different surrogate calibrant levels by plotting peak area ratios of surrogate calibrants and corresponding internal standards against respective response-factor adjusted (see Tables S3 and S4) surrogate calibrant concentrations. Target analyte concentration was calculated via the ratio of the target analyte and its internal standard and the calibration equation of its corresponding surrogate calibrant, respectively. During the measurement of one batch (containing samples of a full 96-well plate) calibration lines were determined at the beginning, middle and end of the sequence. Three QCs, QC_3xLLOQ_, QC_MID_, and QC_HIGH_, were embedded after every calibration and after every 20th sample in the sequence to verify quantitative method performance. Automated integration with the MultiQuant 3.0 software (Sciex) was done using the embedded MQIII algorithm, Gaussian smoothing (width: 1 or 2 data points), noise percentage of 90%, baseline subtraction window of 0.1 min and a peak splitting factor of 2. Excel (Office 365, Version 2006, Microsoft, Redmond, WA, USA), SPSS Statistics 26 (IBM, Armonk, NY, USA), and Origin 2020b (OriginLab, Northampton, MA, USA) were used for further data evaluation. The data visualization and statistics were conducted using Python 3.13.0.

## Results and Discussion

### LC–MS Method

Considering the lower flow limit of the 1290 infinity II system, the flow rate was optimized for the 0.1 mm ID core–shell particle column (Kinetex XB-C18) between 0.05 and 0.1 mL/min. Operating at 0.1 mL/min provided a rapid elution within 9 min, including re-equilibration while maintaining high sensitivity, particularly for critical analytes such as E2 and E3 (Figure S5). Higher flow rates were not pursued, as the detection sensitivity significantly dropped with increasing flow.^49^ Despite the rapid gradient elution, the chromatographic separation of E2 and T with their epimers (epiE2 and epiT) was achieved (Figure S3). E1 and E2 maintained baseline separation even after derivatization (Figure 1). The injection volume was optimized to 10 μL, providing maximal sensitivity without observable overloading. Minimizing extra-column volume is essential to reduce extra-column peak dispersion and prevent band broadening, which can compromise chromatographic resolution and sensitivity.^50^ To address this, an external column oven was strategically installed in close proximity to the ion source inlet. Furthermore, the capillary length between the LC outlet and MS inlet was minimized to 8 cm (corresponding to a volume of 0.9 μL with a 0.12 mm i.d.) via direct connection by circumventing the default grounding unit of the ion source. A specialized grounding cable was employed to prevent additional grounding paths. Those optimizations effectively minimize extra-column volume and preserve peak integrity.

**Figure 1:**
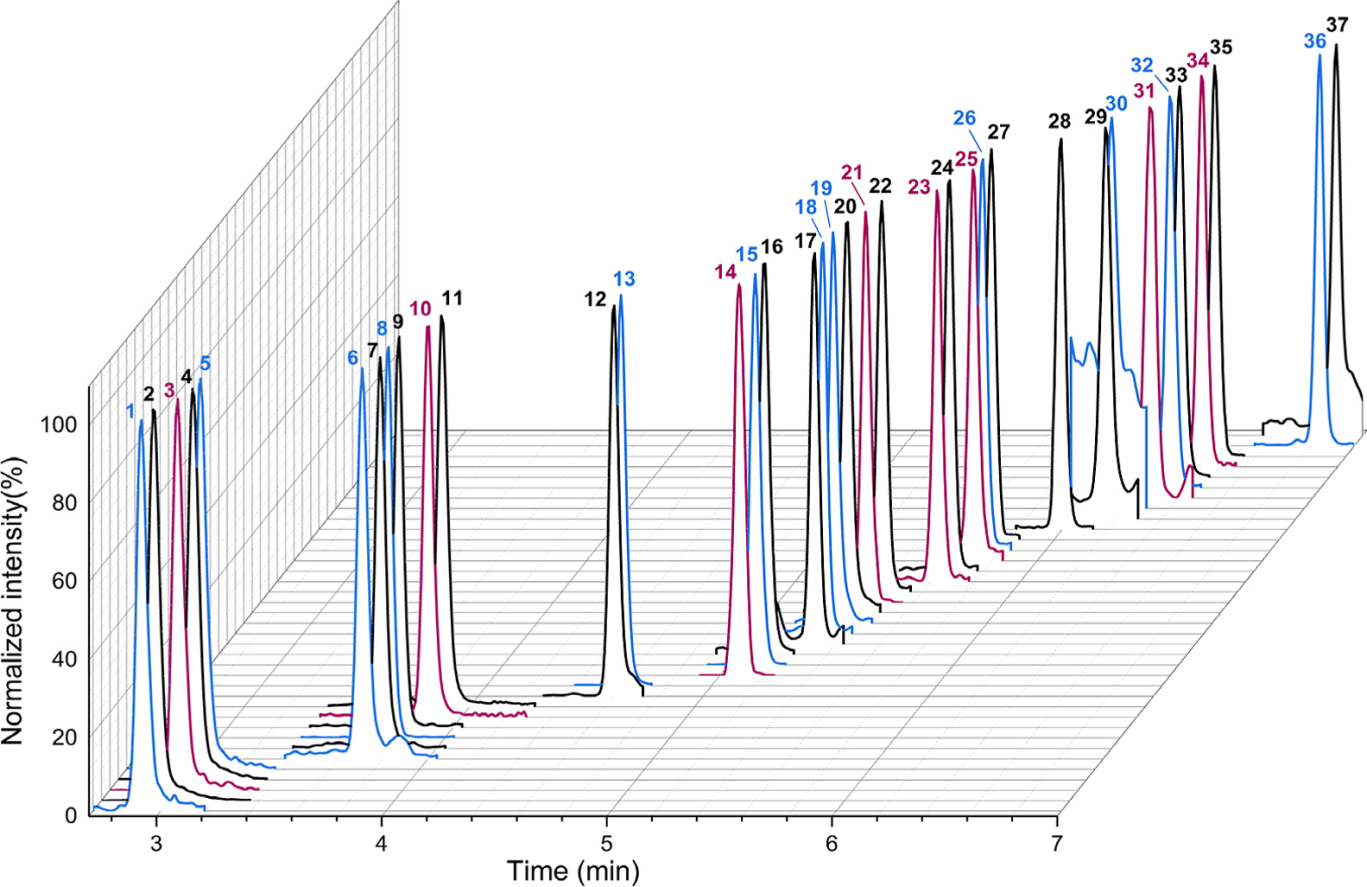
Overlay of normalized chromatograms (quantifier mass transition) of steroids in human plasma. Target analytes are shown in black, surrogate calibrants are marked in blue, and internal standards are highlighted in red. 1: cortisol-*d*_4_; 2: cortisol; 3: cortisone-*d*_8_; 4: cortisone; 5: cortisone-^13^C_3_; 6: CORT-*d*_8_; 7: CORT; 8: E3-*d*_3_ DMIS; 9: E3 DMIS; 10: dienogest-*d*_8_; 11: dienogest; 12: T; 13: T-^13^C_3_; 14: 17OHP-*d*_8_; 15: 17OHP–^13^C_3_; 16: 17OHP; 17: E2 DMIS; 18: E2-^13^C_3_ DMIS; 19: DHT-*d*_3_; 20: DHT; 21: LNG-*d*_6_; 22: LNG; 23: EE2-*d*_4_ DMIS; 24: EE2 DMIS; 25: E1-^13^C_6_ DMIS; 26: E1-^13^C_3_ DMIS; 27: E1 DMIS; 28: NoAc; 29: Preg; 30: Preg-^13^C_2_d_2_; 31: P-*d*_9_; 32: P–^13^C_3_; 33: P; 34: ChAc-*d*_6_; 35: ChAc; 36: ALLO-*d*_5_; 37: ALLO.

Advanced sMRM was employed for data acquisition, ensuring at least two ion transitions for each target analyte, surrogate calibrant, and internal standard. Dwell weight was strategically increased for analytes anticipated at low concentrations in patient samples. Most target compounds were analyzed in positive ion mode to gain higher sensitivity with interference-free acquisition at their corresponding retention times. However, cortisol/cortisol-*d*_4_ and cortisone/cortisone-^13^C_3_ were quantified in negative ion mode due to the interference observed in positive ionization. After automatic tuning of compounds in 50% MeOH with 0.1% formic acid, the 5 most abundant mass transitions were checked for sensitivity and selectivity in the spiked matrix as a preliminary experiment, as background noise and selectivity can differ significantly between neat solution and real sample matrices. Two ion transitions per compound were selected as quantifiers and qualifiers based on their sensitivity and selectivity. Qualifier-to-quantifier ratios were monitored with an acceptance criterion of a maximum ±2 standard deviations (±2σ) compared to the average ratio in calibration runs. Ion source parameters and collision gas pressure were further optimized through systematic on-column injections to ensure robust and reliable performance.

### Sample Preparation

Oasis PRiME HLB SPE in a 96-well plate format was used to enrich and purify target analytes, enabling high-throughput processing. As elution with organic solvent necessitates evaporation prior to RP-UHPLC-MS/MS analysis, an 8 h drying step was implemented. Although lengthy, this evaporation process was fully automated and carried out overnight using a vacuum concentrator (Genevac EZ-2) with inert gas protection. This ensured consistent dryness across wells without impacting daytime analytical throughput. As an alternative, we evaluated a nitrogen blowdown concentrator, which achieved complete evaporation of 600 μL methanol (post-SPE eluent) within 30 min at room temperature in a 96-well plate format. The use of commercially available high-capacity nitrogen blowdown systems may offer a viable path for further increasing throughput and operational efficiency in future implementations.

### Derivatization Conditions

The derivatization step was implemented to enhance the mass spectrometric response of estrogens. DMIS was selected as the derivatization reagent due to its selective reactivity with phenolic hydroxyl groups and its previously reported estrogen-specific fragmentation patterns.^45^ Notably, this study is the first to demonstrate successful DMIS derivatization of ethinylestradiol, extending the applicability of this reagent to synthetic estrogens. The organic content of the derivatization solvent was set to 30% to align with the initial LC condition (25% organic fraction) while ensuring sufficiently reactive conditions. Acetone, ACN, and DMSO at 30% were evaluated for their effectiveness in achieving efficient derivatization and producing favorable peak shapes, particularly for early eluting compounds such as corticosterone, dienogest, and estriol-DMIS. As shown in Figure S4, both acetone and DMSO yielded improved peak shapes for corticosterone and dienogest. However, DMSO proved unsuitable due to insufficient derivatization, as indicated by the absence of the estriol-DMIS peak. Consequently, 30% acetone was selected, and its compatibility with the optimized gradient ensured adequate analyte focusing prior to elution. Derivatization did not diminish the signal intensity of other analytes. However, stability tests (Table S7) revealed that DMIS-derivatized estrogens (E1, E2, E3, EE2) decomposed significantly after 50 h, in sharp contrast to nonderivatized steroids. This outcome suggests that hydrolysis may occur, causing the labeling group to detach from the estrogens and potentially contributing to the observed instability. Despite this limitation, the method remains viable for routine analysis, if all samples in the 96-well plate are analyzed within 22 h. Additionally, the use of IS effectively compensates for any potential losses due to degradation. It is, therefore important that the samples are analyzed soon after derivatization and are not stored (at room temperature) for extended periods. In this study, sufficient stability was found as the 96-well plates were analyzed not later than 2 h after sample preparation.

### Selectivity and Assay Specificity

For each compound, two ion transitions were monitored: one chosen as the quantifier based on superior sensitivity, and the other as a qualifier. Selectivity and specificity were assessed for both transitions by injecting each individual compound at its highest concentration level, including surrogate calibrants, target analytes, and internal standards while monitoring the other transitions. The absence of extracted ion chromatogram (EIC) traces from other compounds confirmed that no cross-talk and interference, respectively, occurred during data acquisition. In addition, epiT and epiE2, epimers of T and E2 with identical fragmentations, were assessed to ensure chromatographic baseline separation (epiT to T, Δ*t*_R_:0.41 min; epiE2-DMIS to E2-DMIS, Δ*t*_R_:0.21 min, see Figure S3.). The chromatographic selectivity of E1 and E2 was also reached after derivatization (E1-DMIS to E2-DMIS, Δ*t*_R_:0.41 min). The absence of interferences for both the surrogate calibrants and the internal standards was confirmed by analyzing six different blank plasma samples, none of which exhibited any detectable peaks at the retention times of those analytes (see Figures S1 and S2).

### Calibration and Limits of Quantification

In the absence of an appropriate blank matrix for matrix-matched calibration, a surrogate calibrant strategy was implemented for endogenous hormones. Due to the limited availability of SIL standards, an ideal experimental design using two SIL compounds with a selective mass shift (typically ≥3 Da) per target analyte was not feasible. Therefore, priority was given to using a SIL analogue as a surrogate calibrant for each corresponding target. When a second SIL analogue was unavailable, internal standards were assigned based on their ability to best control variation (e.g., matrix effects) (Table 1). Here it is worth noting that, due to isotopic interference observed between testosterone-*d*_5_ (T-*d*_5_) and the M + 2 isotopic peak of T-^13^C_3_ on triple quadrupole platforms, T-*d*_5_ could not be used as an internal standard for testosterone quantification (Figure S6). Instead, 17OHP-*d*_8_ was selected based on validation results. Future studies may consider the use of testosterone-*d*_8_ or other alternatives that have recently become available. The concentration of surrogate standards was adjusted to match the response of target standards, and the response factor (RF) of surrogate calibrants and target analytes was balanced via concentration adjustments (applied if RF > 1.10) and detuning of DP and CE parameters (applied if RF < 0.90). As shown in Table S4, the RF in the present case ranged from 0.929 (Preg-^13^C_2_-*d*_2_/Preg) to 1.072 (E3-*d*_3_/E3). To ensure that the surrogate-based quantification is both accurate and representative, the parallelism between the surrogate calibration curve and the target analyte’s standard addition curve must be demonstrated by evaluating the slope ratio (surrogate/target analyte). A range of 95.0–105.0% was considered acceptable. In the present batch, the slope ratio ranged from 96.21 ± 1.02% to 102.28 ± 2.33% (shown in Table S3). Exogenous steroids were quantified by directly spiking the target analytes into pooled male plasma, which was collected for method validation and considered free of contraceptives. Corresponding SIL-IS are specified in Table 1.

**Table 1:** Steroids and Corresponding SIL-IS

steroids	abbr	IS
3α-allopregnanolone	ALLO	ChAc-*d*_6_
Corticosterone	CORT	dienogest-*d*_8_
Cortisol		cortisone-*d*_8_
cortisone		cortisone-*d*_8_
5α-dihydrotestosterone	DHT	LNG-*d*_6_
estrone	E1	E1-^13^C_6_
17β-estradiol	E2	E1-^13^C_6_
estriol	E3	dienogest *d*_8_
pregnenolone	preg	ChAc-*d*_6_
progesterone	P	P-*d*_9_
17α-hydroxyprogesterone	17OHP	17OHP-*d*_8_
17β-testosterone	T	17OHP-*d*_8_
chlormadinone acetate	ChAc	ChAc-*d*_6_
dienogest		dienogest-*d*_8_
ethinylestradiol	EE2	EE2-*d*_4_
levonorgestrel	LNG	LNG-*d*_6_
nomegestrol acetate	NoAc	ChAc-*d*_6_

LODs and LOQs were determined using a 15-point calibration in the matrix (1:1 serial dilution). The resulting LLOQs and ULOQs were selected to cover reference ranges in population. Cortisol/cortisol-*d*_4_ and cortisone/cortisone-^13^C_3_ were quantified in negative ion mode to circumvent interferences encountered under positive ionization mode. Although lower LLOQs would have been achieved via the [M + HCOO]^−^ precursor ions,^51^ the [M – H]^−^ adducts were chosen as they provided a linear range better aligned with the expected physiological concentrations of these analytes in plasma.

### Matrix Effect, Extraction Recovery, Process Efficiency, Accuracy, and Precision

Extraction recovery (ER), matrix effect (ME), and process efficiency (PE) were assessed following the approach described by Matuszewski et al.^52^ with labeled surrogate calibrants, which are expected to show equal results to coeluted target analytes. Briefly, five different plasma lots were measured in triplicate at three QC levels: QC_3xLLOQ_, QC_MID_, and QC_HIGH_. In the neat solution and pre/post-spiking experiments, adjusted amounts of surrogate calibrant and target analyte mixtures were spiked at three QC levels. Additionally, equivalent amounts of internal standards were spiked before extraction simultaneously to determine whether normalization using internal standards could compensate for ER, ME, and PE variations. As shown in Table S5, when corrected by internal standards, ER ranged from 73.8% (E3-*d*_3_) to 111.7% (P–^13^Cd_3_), while ME ranged from 76.5% (ionization suppression for Preg-^13^C_2_-*d*_2_) to 110.6% (ionization enhancement for E1-^13^C_3_). Those results indicate the critical role of internal standards in correcting matrix-related variability and ensuring more reliable quantification.

Intra-assay and interday precision and accuracy were assessed in plasma using surrogate calibrants, following the FDA guideline for bioanalytical method validation. Four QC levels (QC_LLOQ_, QC_3xLLOQ_, QC_Mid_, and QC_HIGH_) were analyzed in quintuplicate (*n* = 5) across three separate days. Detailed results are presented in Table S6. Precisions were below 15%, and accuracies ranged from 85% to 115% throughout the entire range, including at the LLOQ level. These confirm that the assay provides adequate specificity and reliable quantification under the current experimental conditions.

The method’s performance was further verified through cross-validation using external and certified quality control samples, including the MassCheck Steroid Panel 2, NIST SRM 1950, BCR 576, BCR 577, and BCR 578. The results are shown in Table 2.

**Table 2:** Validation via External, Certified Quality Controls^a^

type	analyte	target conc. [pg/mL]	target range [pg/mL]	prec. [%]	acc. [%]
MassCheck steroid panel 2 level I (*n* = 8)	DHT	83	58–108	2.8	103.2
	E2	82	57–107	12.2	95.9
	P	310	217–403	2.4	93.2
	17OHP	300	210–390	15.6	101.6
	T	201	141–261	7.3	79.0
MassCheck steroid panel 2 level II (*n* = 8)	DHT	368	294–442	1.4	110.7
	E2	411	329–493	6.3	98.8
	P	3180	2540–3810	5.5	88.1
	17OHP	1540	1230–1840	14.8	101.8
	T	1520	1210–1820	6.2	86.9
MassCheck steroid panel 2 level III (*n* = 8)	DHT	1050	842–1260	2.0	113.0
	E2	2500	2000–3000	7.1	97.7
	P	15,100	12,100–18,200	4.7	91.1
	17OHP	8960	7170–10,700	16.9	100.7
	T	7820	6260–9380	12.7	93.2
NIST SRM 1950 (*n* = 8)	cortisol	83,900	82,200–85,600	5.1	113.8
	P	1482	1444–1520	6.3	95.2
	T	2214	2167–2261	8.2	92.8
BCR 576 (*n* = 4)	E2	31.1	29.7–32.4	6.4	120.4
BCR 577 (*n* = 4)	E2	188	177–199	9.5	98.1
BCR 578 (*n* = 4)	E2	365	346–384	5.0	107.1

a For MassCheck steroid panel 2 controls, level I T did not achieve ±15% bias but accuracy was still in the reported product target range (mean concentration: 158.8 pg/mL).

### Autosampler Stability

The stability of the analytes, stored at 4 °C in the autosampler after sample preparation, was evaluated after 12 and 50 h. For this assessment, surrogate calibrants and analytes were spiked into pooled plasma at three different concentration levels (QC_3xLLOQ_, QC_MID_, QC_HIGH_), each in four replicates. Although DMIS-derivatized compounds were relatively less stable compared to other analytes, their stability remained within acceptable limits after 12 h, particularly when internal standardization was applied (see Table S7). To maintain accurate quantification of derivatized estrogens, recalibration after 12 h is recommended, especially for compounds which do not have a labeled analogue as internal standards (e.g., E2 and E3).

### Application

In a clinical study, the impact of hormonal fluctuations on stress levels in females using hormonal contraceptives was investigated. A total of 86 females were recruited and categorized into three groups: females using LNG-releasing intrauterine devices (IUD, *n* = 27), females using oral contraceptives (OC, *n* = 30), and females with a natural menstrual cycle (NC, *n* = 29). Seventy-five of the eighty-six participants completed two study visits to assess intraindividual variability. Eventually, 163 plasma samples were successfully measured with the validated LC–MS/MS methods. In this clinical measurement, in approximately one-third of the samples, concentration of ALLO and E2 were below the LLoQ (35.6 pg/mL and 3.45 pg/mL, respectively), preventing accurate quantification of these steroids. Therefore, a more sensitive method is needed specifically for ALLO and E2 quantification. Regarding E3, only around 20% of the sample had concentrations above the LLoQ (1.07 pg/mL), which was expected given that E3 is primarily present in significant levels during pregnancy.

The determined concentrations of the targets are illustrated in Figure 2 using box plots. For most endogenous hormones, including Preg, ALLO, P, 17OHP, E1, and E2, NC females exhibited the highest concentrations, followed by IUD and OC users (all *p* < 0.00135, Kruskal–Wallis test, applicable to the following results). Similarly, for DHT, NC females had higher concentrations than the other two groups (*p* = 0.00067). In contrast, the stress hormones cortisol and cortisone followed an inverse pattern, with OC users showing significantly higher concentrations compared to NC and IUD groups (*p* < 3.40 × 10^–8^). Statistical analysis of targets is summarized in Table S8. Exogenous hormones detected in the samples were LNG for IUD users and EE in combination with a progestin-most commonly LNG-for OC users. A detailed discussion and interpretation of the results is available in Bürger’s work.^53^

**Figure 2:**
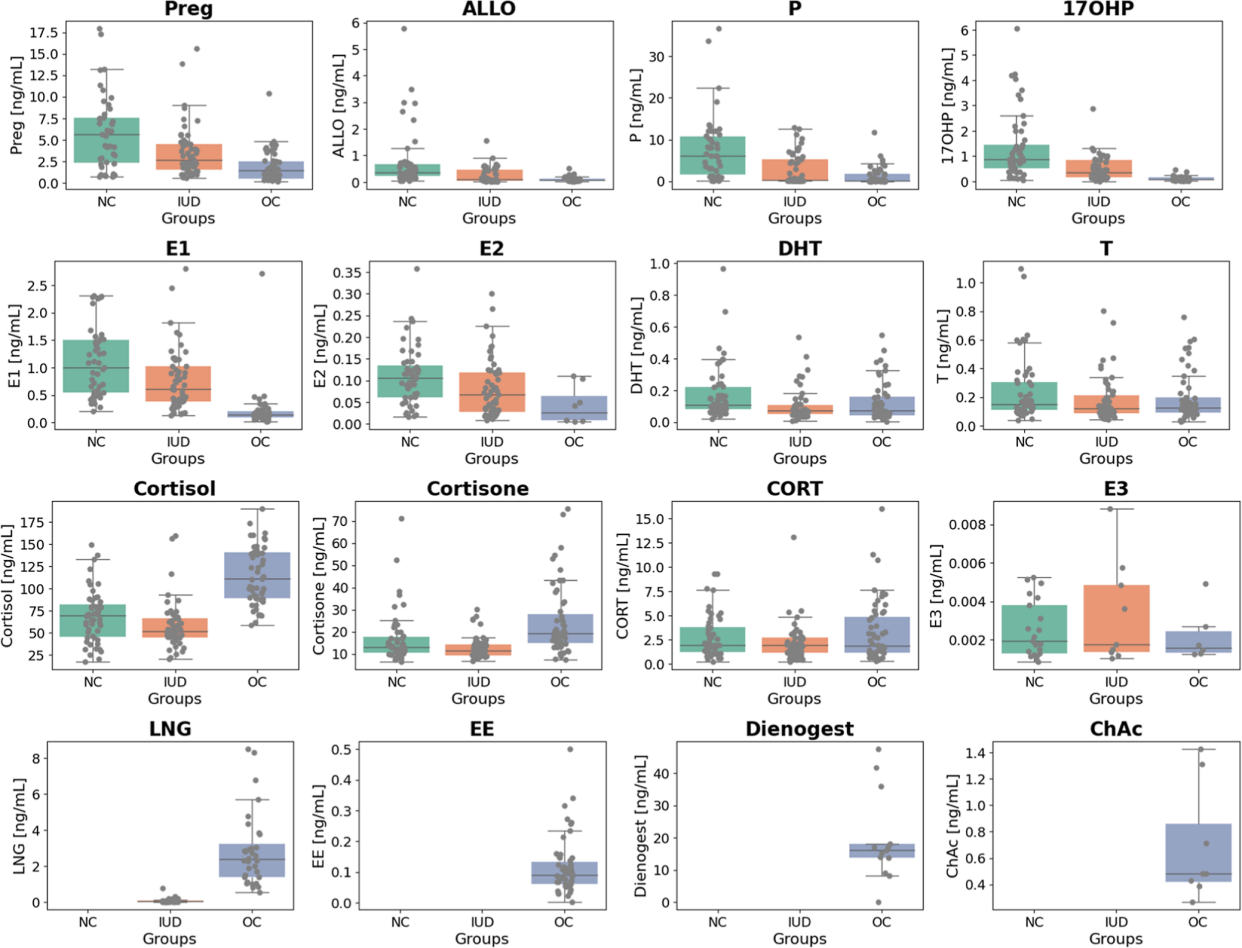
Boxplot distribution of endogenous and exogenous steroid concentrations in samples collected from females with a natural menstrual cycle (NC), using intrauterine devices (IUD), and using oral contraceptives (OC).

## Conclusions

This study presented a high-throughput LC–MS/MS method for steroid quantification, utilizing 96-well plate SPE for streamlined sample preparation and achieving a total analysis time of 9 min, which makes it well-suited for large-scale clinical studies. While the current vacuum evaporation step may limit applicability in time-sensitive clinical settings, it was demonstrated that nitrogen-based alternatives offer a practical solution to enhance throughput.

Sensitivity was significantly enhanced by performing low-flow (0.1 mL/min) LC separation on a narrow-diameter (1.0 mm) core–shell C18 column, optimizing ionization efficiency. Furthermore, DMIS-based selective derivatization was employed to improve estrogen detection sensitivity while preserving the integrity of other steroids. A broad steroid panel was achieved through sMRM, allowing simultaneous quantification of derivatized estrogens and nonderivatized steroids in a single analytical run. Accurate and reliable quantification was ensured through the use of ^13^C-labeled SIL surrogate calibrants and deuterated internal standards, effectively correcting for matrix effects and extraction variability. To our knowledge, this study is the first to report and demonstrate the feasibility of combining surrogate calibration with chemical derivatization and features a unique hybrid calibration strategy to maintain analytical validity across endogenous and exogenous target analyte classes without requiring separate analytical runs.

Method validation was performed following FDA bioanalytical method validation guidelines and supplemented with additional criteria to address the lack of formal regulatory guidance, providing a reference for future surrogate calibrant-based quantification studies. Along these lines, validation was complemented by confirming accurate quantification of certified commercial quality controls.

Further improvements in method performance are expected with increased availability of SIL standards, ideally ^13^C-labeled analogues, enabling even more precise quantification through optimum internal standardization.

Overall, this method integrates high sensitivity, efficient sample preparation, and a broad analytical scope, making it a powerful tool for large-scale steroid analysis in clinical and biomedical research. Future advancements in chromatography and next-generation mass spectrometers with enhanced sensitivity will further push analytical limits, enabling steroid research in challenging sample types and lower volumes (e.g., murine plasma).

## Supplementary Material



## Notes

Details on materials, preparation of stock solutions, calibration, and quality controls; tables of calibrant concentrations, sMRM method parameters, response factors, matrix effect, extraction recovery, process efficiency, precision, accuracy, and stability data; steroid profiles in female plasma samples; representative chromatograms of targets, surrogate calibrants, and internal standards in human plasma; epimer separation; optimization of derivatization solvent and flow rate; isotopic interference between T-*d*_5_ and T-^13^C_3_ (PDF)
