# Supplementary material for: Quantification of Endogenous Steroids and Hormonal Contraceptives in Human Plasma via Surrogate Calibration and UHPLC-MS/MS
Source: Anal Chem. 2025 Jun 20;97(25):13496–503. doi: 10.1021/acs.analchem.5c01912 (PMC12224168; doi:10.1021/acs.analchem.5c01912)
Supplement: Supplementary file 1 [file ac5c01912_si_001.pdf]

Supporting Information

Quantification of Endogenous Steroids and Hormonal Contraceptives in Human Plasma via Surrogate Calibration and UHPLC-MS/MS

Min Su[1], Bernhard Drotleff[2], Tamara Janker[1], Zoé Bürger[3,4], Ann-Christin S. Kimmig[3,] , Birgit Derntl[3,5], Michael Lämmerhofer[1]*

[1]Institute of Pharmaceutical Sciences, Pharmaceutical (Bio-)Analysis, University of Tuebingen, Tuebingen, Germany

[2]Metabolomics Core Facility, EMBL Heidelberg, Heidelberg, Germany

[3]Department of Psychiatry and Psychotherapy, Tübingen Center for Mental Health (TüCMH), University of Tübingen, Tübingen, Germany

[4]Department of Women's and Children's Health, Science for Life Laboratory, Uppsala University, Uppsala, Sweden

[5]LEAD Research School and Graduate Network, University of Tuebingen, Tuebingen, Germany

Author for correspondence:

Dr. Bernhard Drotleff

Metabolomics Core Facility

EMBL Heidelberg

Meyerhofstraße 1

69117 Heidelberg, Germany

T: +49 6221 387-8705

e-mail: bernhard.drotleff@embl.de

www.embl.org/groups/metabolomics

Contents

Materials ······ S3

Preparation of stock solutions, calibrants, and quality controls ······ S5

LC-MS method ······ S6

Table S1. MS method-parameters for advanced sMRM ······ S7

Table S2. Surrogate calibrant concentration in pg/mL ······ S10

Table S3. Method properties ······ S11

Table S4. Response factors of surrogate calibrants and target analytes in neat solution after balancing via concentration (if >10% deviation) and DP / CE (<10% deviation) ······ S13

Table S5. Matrix effect, extraction recovery, process efficiency ······ S14

Table S6. Validation results of precision and accuracy ······ S16

Table S7. Post-processing stability ······ S18

Table S8. Steroid profiles and reference intervals for endogenous hormones ······ S20

Fig. S1. Extracted ion chromatograms of steroids ······ S21

Fig. S2. Extracted ion chromatograms of internal standards in blank and spiked matrix ······ S23

Fig. S3. Chromatographic selectivity for steroid epimers ······ S24

Fig. S4. Optimization of derivatization solvents ······ S26

Fig. S5. Flow optimization ······ S27

Fig. S6. Isotopic interference between T-d_5 and the M+2 peak of T-$^{13}C_3$ ······ S28

Reference ······ S29

Materials

Acetone (HPLC grade, Cat. No. 270725), chlormadinone acetate (ChAc, Cat. No. Y0001653), cortisone (Cat. No. C2755), cortisone-2,3,4-$^{13}C_3$ (100 µg/mL in methanol, Cat. No. C-160), dienogest (Cat. No. Y0001785), 5α-dihydrotestosterone (DHT, Cat. No. 10300), 5α-dihydrotestosterone-16,16,17-$^{2}H_3$ (DHT-d_3, 100 µg/mL in methanol, Cat. No. D-077), dimethyl sulfoxide (DMSO, Cat. No. 34869), ethinylestradiol (EE2, Cat. No. E1900000), 17β-estradiol (E2, Cat. No. 3301), 17β-estradiol-2,3,4-$^{13}C_3$ (E2-$^{13}C_3$, Cat. No. 719552), 17α-estradiol (epiestradiol, epiE2, Cat. No. 46542), 17α-hydroxyprogesterone (17OHP, Cat. No. H5752), 17α-hydroxyprogesterone-2,3,4-$^{13}C_3$ (17OHP-$^{13}C_3$, 100 µg/mL in methanol, Cat. No. H-100), levonorgestrel (LNG, Cat. No. L0551000), nomegestrol acetate (NoAc, Cat. No. N1080000), pregnenolone-20,21-$^{13}C_2$-16,16-$^{2}H_5$ (Preg-$^{13}C_2$-d_2, Cat. No. 739545), sodium hydrogen carbonate (Cat. No. S5761), sodium carbonate (Cat. No. 223484), 17β-testosterone (T, Cat. No. 86500), 17β-testosterone-2,3,4-$^{13}C_3$ (T-$^{13}C_3$, 100 µg/mL in methanol, Cat. No. T-070), 17α-testosterone (epitestosterone, epiT, 1.0 mg/mL in acetonitrile, Cat. No. E-058), and zinc sulfate heptahydrate ($ZnSO_4$, Cat. No. 221376) were purchased from Sigma Aldrich (Merck, Darmstadt, Germany). Progesterone-2,3,4-$^{13}C_3$ (P-$^{13}C_3$, Cat. No. 10314) and estrone-2,3,4-$^{13}C_3$ (E1-$^{13}C_3$, 100 µg/mL in methanol, Cat. No. S9125) were purchased from IsoSciences (King of Prussia, PA, USA). 17α-Hydroxyprogesterone-2,2,4,6,6,21,21,21-$^{2}H_8$ (17OHP-d_8, Cat. No. DLM-6598), estrone-13,14,15,16,17,18-$^{13}C_6$ (E1-$^{13}C_6$, Cat. No. CLM-7935), and cortisol (Cat. No. ULM-9141) were obtained from Euriso-Top GmbH (Saarbrücken, Germany). Corticosterone (CORT, Cat. No. 16063), estriol (E3, Cat. No. 10006484), estrone (E1, Cat. No. 10006485), and progesterone (P, Cat. No. 15876) were purchased from Cayman Chemical (Ann Arbor, MI, USA). 3α-Allopregnanolone (ALLO, Cat. No. A547100), 3α-allopregnanolone-2,2,3,4,4-$^{2}H_5$ (ALLO-d_5,

Cat. No. A547102), corticosterone-2,2,4,6,6,17,21,21-$^2H_8$ (major, CORT-d_8, Cat. No. C695702), cortisol-9,11,12,12-$^2H_4$ (cortisol-d_4, Cat. No. C696302), cortisone-2,2,4,6,6,9,21,21-$^2H_8$ (major, cortisone-d_8, Cat. No. C696502), dienogest-2,2,4,8,11,11,19,19-$^2H_8$ (major, dienogest-d_8, Cat. No. D441873), estriol-2,4,17-$^2H_3$ (E3-d_3, Cat. No. E888962), ethinylestradiol-2,4,16,16-$^2H_4$ (EE2-d_4, Cat. No. E685102), levonorgestrel-2,2,4,6,6,10-$^2H_6$ (LNG-d_6, Cat. No. N689513), pregnenolone (Preg, Cat. No. P712200), and progesterone-2,2,4,6,6,17,21,21,21-$^2H_9$ (P-d_9, Cat. No. P755902) were acquired from Toronto Research Chemicals (North York, ON, Canada). Chlormadinone acetate-2,2,4,23,23,23-d6 (ChAc-d_6, Cat. No. HY-B1095S1) was purchased from MedChemExpress (Monmouth Junction, NJ, USA). 1,2-Dimethylimidazole-5-sulfonyl chloride (DMIS, Cat. No. OR2145) for derivatization was provided by Apollo Scientific (Stockport, UK). Methanol (MeOH, Ultra LC-MS grade, Cat. No. HN41), acetonitrile (MeCN, Ultra LC-MS grade, HN40), and formic acid (≥98%, w/v, Cat. No. 4724) were supplied by Carl Roth (Karlsruhe, Germany). Type I purity water was obtained from a Purelab Ultra purification system (ELGA LabWater, Celle, Germany). The certified reference materials NIST SRM 1950, BCR 576, BCR 577, and BCR 578 were purchased from Sigma Aldrich (Merck), MassCheck Steroid Panel 2 (tri-level) was acquired from Chromsystems (Graefelfing, Germany).

Preparation of stock solutions, calibrants, and quality controls

Preparation of 7 calibrants and 4 QCs (QC_{LLOQ}, QC_{3xLLOQ}, QC_{MID}, and QC_{HIGH}). Concentrations see Table S1. Stock solutions of solid reference standards were prepared in MeOH at 1.0 mg/mL. Instrument responses of equimolar mixtures of target analytes and surrogate calibrants were checked and if the deviation was >10%, the surrogate calibrant concentration was adjusted to match the instrument response of the target analyte. Fine tuning of instrument response balancing was done via changes in DP or CE of the surrogate calibrant. Using the adjusted stock solutions, two master mixes containing all target analytes and surrogate calibrants were prepared. Master mix high was used to prepare QC_{HIGH}, calibrant 5, 6, and at ULOQ, master mix low was used to prepare QC_{LLOQ}, QC_{3xLLOQ}, QC_{MID}, calibrant 4, 3, 2, and at LLOQ. Prior to every sequence, master mixes were diluted, dried and derivatized and the response factor of each target analyte-surrogate calibrant pair was determined. For quantification, surrogate calibrant concentrations of calibrants and QCs were adjusted via the corresponding response factor. A plasma pool of 10 different male donors was used as matrix for calibrants and QCs (analyte-free for contraceptives).

LC-MS method

Mobile phase A consisted of H2O + 0.1% formic acid (v/v) and mobile phase B of MeCN + 0.1% formic acid (v/v). The following gradient was applied: 25 - 85% B from 0.0 – 6.5 min, 85 – 95% B from 6.5 – 6.6 min, constant 95% B from 6.6 – 7.2 min, 95 – 25% B from 7.2 – 7.3 min, re-equilibration at 25% B from 7.3 – 9.0 min. The flow rate during analysis was set to 0.1 mL/min and was increased to 0.125 mL/min during the flushing and re-equilibration period from 6.6 – 8.5 min to minimize overall run time. Injection volume was set to 10 µL and MeOH was used as needle wash.

Optimized mass spectrometer ion source parameters were: curtain gas (N_2) 45 psi; nebulizer gas (zero air) 30 psi; heater gas (zero air) 30 psi; ion spray voltage 5500 V in positive and -4500 V in negative ionization mode; entrance potential 10 V in positive and -10 V in negative ionization mode; medium collision gas (N_2) pressure; ion source temperature 450 °C. Samples were measured in advanced sMRM mode. Total cycle time (t_{Cyc}) was delimited to 500 ms to attain at least twelve data points per peak in regard to average peak widths of about 6 s.

Table S1. MS method-parameters for advanced sMRM.[a]

Analyte	Sum formula	Q1 precursor mass [*m/z*]	Q3 fragment mass [*m/z*]	RT window [min]	DP [V]	CE [V]	CXP [V]	Dwell weight
ALLO	$C_{21}H_{34}O_2$	301.3	135.1	6.90 ± 0. 30	106	25	12	2.0
		301.3	283.2		106	15	12	
ALLO-d$_5$	$C_{21}H_{29}{}^2H_5O_2$	306.3	140.2	6.85 ± 0.25	111	21	10	
		306.3	288.3		111	17	12	
CORT	$C_{21}H_{30}O_4$	347.3	121.1	3.73 ± 0.30	91	27	10	1.0
		347.3	329.2		91	21	14	
CORT-d$_8$	$C_{21}H_{22}{}^2H_8O_4$	355.3	125.1	3.71 ± 0.30	96	29	10	
		355.3	337.3		96	21	14	
Cortisol	$C_{21}H_{30}O_5$	361.2	297.1	2.93 ± 0.50	-90	-32	-11	2.0
		361.2	282.0		-90	-53	-10	
Cortisol-d$_4$	$C_{21}H_{26}{}^2H_4O_5$	365.2	301.1	2.91 ± 0.50	-97	-34	-11	
		365.2	286.0		-90	-53	-10	
Cortisone	$C_{21}H_{28}O_5$	359.2	301.1	3.00 ± 0.50	-70	-20	-11	2.0
		359.2	136.9		-70	-39	-9	
Cortisone-$^{13}C_3$	$C_{18}{}^{13}C_3H_{28}O_5$	362.2	304.1	3.00 ± 0.50	-90	-20	-11	
		362.2	136.9		-90	-39	-9	
Cortisone-d$_8$[#]	$C_{21}H_{20}{}^2H_8O_5$	367.2	307.2	3.00 ± 0.50	-75	-20	-11	0.5
		367.2	138.0		-75	-40	-9	0.2
DHT	$C_{19}H_{30}O_2$	291.2	255.2	5.34 ± 0.15	91	21	10	2.0
		291.2	91.0		91	65	14	
DHT-d$_3$	$C_{19}H_{27}{}^2H_3O_2$	294.3	258.2	5.33 ± 0.15	102	21	10	
		294.3	91.0		86	71	10	
E1	$C_{18}H_{22}O_2$	429.2	365.2	5.71 ± 0.15	31	41	14	1.0
		429.2	96.1		31	65	14	
E1-$^{13}C_3$	$C_{15}{}^{13}C_3H_{22}O_2$	432.2	368.2	5.72 ± 0.15	24	40	14	
		432.2	96.1		24	65	14	
E1-$^{13}C_6$[#]	$C_{12}{}^{13}C_6H_{22}O_2$	435.2	371.2	5.72 ± 0.15	11	39	14	0.5
		435.2	96.1		11	63	14	0.2
E2	$C_{18}H_{24}O_2$	431.2	367.2	5.31 ± 0.15	20	39	14	2.0
		431.2	96.1		20	69	14	
E2-$^{13}C_3$	$C_{15}{}^{13}C_3H_{24}O_2$	434.2	370.3	5.30 ± 0.15	20	39	14	

		434.2	96.1		20	67	14	
E3	$C_{18}H_{24}O_3$	447.2	383.2	3.75 ± 0.30	16	41	14	2.0
		447.2	96.1		16	65	14	
E3-d$_3$	$C_{18}H_{21}{}^2H_3O_3$	450.2	386.3	3.75 ± 0.30	16	47	14	
		450.2	96.1		16	69	14	
Preg	$C_{21}H_{32}O_2$	299.2	91.0	6.15 ± 0.15	101	67	8	2.0
		299.2	105.0		101	65	10	
Preg-$^{13}C_2$-d$_2$	$C_{19}{}^{13}C_2H_{30}{}^2H_2O_2$	303.1	91.0	6.14 ± 0.15	101	53	8	
		303.1	105.0		101	55	8	
P	$C_{21}H_{30}O_2$	315.2	97.1	6.34 ± 0. 20	91	25	10	2.0
		315.2	109.1		91	27	8	
P-$^{13}C_3$	$C_{18}{}^{13}C_3H_{30}O_2$	318.2	100.1	6.33 ± 0.20	91	25	10	
		318.2	112.1		91	29	8	
P-d$_9$#	$C_{21}H_{21}{}^2H_9O_2$	324.3	100.1	6.28 ± 0.20	101	27	10	0.5
		324.3	113.1		101	29	8	0.2
17OHP	$C_{21}H_{30}O_3$	331.2	97.1	5.12 ± 0.20	71	25	8	2.0
		331.2	109.1		71	29	8	
17OHP -$^{13}C_3$	$C_{18}{}^{13}C_3H_{30}O_3$	334.2	100.1	5.11 ± 0.20	91	27	8	
		334.2	112.1		107	31	8	
17OHP -d$_8$#	$C_{21}H_{22}{}^2H_8O_3$	339.3	100.1	5.09 ± 0.20	91	27	10	0.5
		339.3	113.1		91	31	8	0.2
T	$C_{19}H_{28}O_2$	289.2	109.1	4.60 ± 0.20	91	27	8	2.0
		289.2	97.1		91	25	8	
T-$^{13}C_3$	$C_{18}{}^{13}C_3H_{30}O_3$	292.2	112.1	4.60 ± 0.15	108	29	10	
		292.2	100.1		91	25	10	
ChAc	$C_{23}H_{29}ClO_4$	405.2	345.2	6.42 ± 0.15	96	17	14	1.0
		405.2	309.2		96	21	12	
ChAc-d$_6$#	$C_{23}H_{23}{}^2H_6ClO_4$	411.2	351.2	6.40 ± 0.15	81	19	14	0.5
		411.2	315.2		81	23	12	0.2
Dienogest	$C_{20}H_{25}NO_2$	312.2	161.1	3.87 ± 0.45	56	33	8	1.0
		312.2	135.0		56	37	12	
Dienogest-d$_8$#	$C_{20}H_{17}{}^2H_8NO_2$	320.3	167.1	3.85 ± 0.45	56	35	12	0.5
		320.3	139.1		56	39	10	0.2
EE2	$C_{20}H_{24}O_2$	455.2	391.2	5.64 ± 0.20	26	41	15	2.0
		455.2	96.1		26	80	14	

EE2-d_4[#]	$C_{20}H_{20}{}^2H_4O_2$	459.2	395.3	5.63 ± 0.20	26	41	10	0.5
		459.2	96.1		26	69	16	0.2
LNG	$C_{21}H_{28}O_2$	313.2	245.2	5.41 ± 0.20	96	23	10	1.0
		313.2	109.1		96	29	8	
LNG-d_6[#]	$C_{21}H_{22}{}^2H_6O_2$	319.3	251.2	5.39 ± 0.03	106	25	10	0.5
		319.3	91.1		106	65	6	0.2
NoAc	$C_{23}H_{30}O_4$	371.2	311.2	5.99 ± 0.15	66	17	12	1.0
		371.2	251.0		66	27	10	

[a]Target analytes optimized, surrogate calibrants deoptimized via DP or CE if balancing of instrument response was required.

[#]Internal standards.

Values outside ± 2σ of the average qualifier transition 2-to-quantifier transition 1 ratio indicate potential interferences.

Table S2. Surrogate calibrant concentration in pg/mL

Analyte	Cal 1	Cal 2	Cal 3	Cal 4	Cal 5	Cal 6	Cal 7	$QC3_{xLLOQ}$	QC_{MID}	QC_{HIGH}
ALLO-d_5	35.6	71.3	106.9	320.8	891.0	3564	14,256	89.1	499.0	4990
CORT-d_8	61.9	123.7	185.6	556.8	1933	7733	30,933	154.7	1083	10,827
Cortisol-d_4	788.0	1576	2364	7092	12,313	49,252	197,009	1970	6895	68,953
Cortisone-$^{13}C_3$	753.7	1507	2261	6783	5888	23,553	94,211	1884	3297	32,974
DHT-d_3	19.4	38.9	58.3	174.9	607.2	2429	9715	48.6	340.0	3400
E1-$^{13}C_3$	1.9	3.8	5.7	17.1	177.8	711.4	2845	4.7	99.6	995.9
E2-$^{13}C_3$	3.5	6.9	10.4	31.1	323.7	1295	5179	8.6	181.3	1813
E3-d_3	1.1	2.1	3.2	9.6	201.0	804.0	3216	2.7	112.6	1126
Preg-$^{13}C_2$-d_2	125.4	250.9	376.3	929.2	1162	4646	18,583	313.6	650.4	6504
P-$^{13}C_3$	1.0	1.9	2.9	8.6	2979	11,914	47,657	2.4	1668	16,680
17OHP -$^{13}C_3$	6.1	12.3	18.4	55.2	766.1	3065	12,258	15.3	429.0	4290
T-$^{13}C_3$	2.1	4.3	6.4	19.2	666.2	2665	10,658	5.3	373.0	3730
ChAc	10.0	20.0	30.0	90.0	1250	5000	20,000	25.0	700.0	7000
Dienogest	50.0	100.0	150.0	450.0	3125	12,500	50,000	125.0	1750	17,500
EE2	2.0	4.0	6.0	18.0	187.5	750.0	3000	5.0	105.0	1050
LNG	10.0	20.0	30.0	90.0	1250	5000	20,000	25.0	700.0	7000
NoAc	20.0	40.0	60.0	180.0	1250	5000	20,000	50.0	700.0	7000

QC_{LLOQ} is not shown as it is also represented by calibrant 1. Concentrations are response factor adjusted and in pg/mL

Table S3. Method properties

Surrogate Calibrant	LLOQ [pg/mL]	ULOQ [pg/mL]	Slope	R^2	Weight-ing	Smooth-ing	LOD [pg/mL]	LOQ [pg/mL]	Slope ratio [%]	Conc. in pool [pg/mL]
ALLO-d_5	35.6	14256	0.00046 ± 0.00010	0.9930 ± 0.0017	$1/x^2$	1	4.83	14.63	102.28 ± 2.33	43.5 ± 6.6
CORT-d_8	61.9	30933	0.00144 ± 0.00002	0.9968 ± 0.0007	1/x	2	14.19	42.99	96.21 ± 1.02	5135 ± 147
Cortisol-d_4	788.0	197009	0.00006 ± 0.00001	0.9900 ± 0.0009	$1/x^2$	2	231.5	701.5	99.63 ± 0.29	126018 ± 5059
Cortisone-$^{13}C_3$	753.7	94211	0.00016 ± 0.00001	0.9918 ± 0.0007	$1/x^2$	2	203.9	617.9	97.9 ± 1.68	18785 ± 1801
DHT-d_3	19.4	9715	0.00480 ± 0.00016	0.9926 ± 0.0033	$1/x^2$	1	3.58	10.86	97.42 ± 0.89	403.6 ± 26.5
E1-$^{13}C_3$	1.9	2845.4	0.01268 ± 0.00029	0.9932 ± 0.0041	1/x	2	0.56	1.68	98.9 ± 2.58	39.0 ± 2.2
E2-$^{13}C_3$	3.45	5179.13	0.00855 ± 0.00149	0.9901 ± 0.0035	$1/x^2$	1	0.77	2.34	99.8 ± 1.94	51.9 ± 3.3
E3-d_3	1.07	3215.95	0.01959 ± 0.00202	0.9865 ± 0.0033	$1/x^2$	1	0.30	0.91	97.65 ± 1.27	0.29 ± 0.36
Preg-$^{13}C_2$-d_2	125.4	18583	0.00016 ± 0.00007	0.9923 ± 0.0075	1/x	1	40.59	123.0	96.22 ± 1.3	2911 ± 280
P-$^{13}C_3$	1.0	47657	0.00390 ± 0.00021	0.9928 ± 0.0006	$1/x^2$	1	0.34	1.03	99.08 ± 0.51	65.5 ± 6.0
17OHP -$^{13}C_3$	6.1	12258	0.00619 ± 0.00165	0.9901 ± 0.0017	$1/x^2$	2	1.02	3.10	97.97 ± 0.46	544.2 ± 22.8
T-$^{13}C_3$	2.13	10658	0.01235 ± 0.00113	0.9884 ± 0.0017	$1/x^2$	1	0.56	1.69	97.02 ± 2.05	3358 ± 131
ChAc	20.0	20000	0.00145 ± 0.00001	0.9989 ± 0.0010	1/x	1	1.82	5.52	-	-
Dienogest	50.0	50000	0.00562 ± 0.00009	0.9976 ± 0.0010	1/x	1	9.32	28.25	-	-
EE2	2.0	3000	0.00334 ± 0.00020	0.9972 ± 0.0022	1/x	2	0.62	1.87	-	-
LNG	10.0	20000	0.00256 ± 0.00005	0.9977 ± 0.0011	1/x	2	2.15	6.50	-	-
NoAc	20.0	20000	0.00633 ± 0.00066	0.9898 ± 0.0053	$1/x^2$	2	1.55	4.70	-	-

LODs and LOQs were calculated via error of regression of a matrix-matched 5-point calibration (no weighting, concentration range: calibrant 5 ≙ 16x calibrant 1). Slope ratio calculation: surrogate calibrant slope/ target analyte slope (standard addition). Gaussian smoothing factor was applied prior to peak integration in MultiQuant software. Background concentration in sample pool was determined via standard addition (extrapolation) during assessment of parallelism.

Table S4. Response factors of surrogate calibrants and target analytes in neat solution after balancing via concentration (if >10% deviation) and DP / CE (<10% deviation)

**Surrogate Calibrant**	**RF**
ALLO-d_5	1.017
CORT-d_8	1.031
Cortisol-d_4	0.942
Cortisone-$^{13}C_3$	0.985
DHT-d_3	0.971
E1-$^{13}C_3$	0.948
E2-$^{13}C_3$	0.971
E3-d_3	1.072
Preg-$^{13}C_2$-d_2	0.929
P-$^{13}C_3$	0.953
17OHP -$^{13}C_3$	1.018
T-$^{13}C_3$	0.967

RF is calculated as the ratio of surrogate calibrant / target analyte for the quantifier mass transitions. Determined on 2 different levels (master mix high, master mix low) in triplicate per level.

Table S5. Matrix effect, extraction recovery, process efficiency.[b]

Analyte	QC level	RE [%]		ME [%]		PE [%]	
		Peak area	IS ratio	Peak area	IS ratio	Peak area	IS ratio
**ALLO-d_5**	QC_{3xLLOQ}	64.4 ± 11.1	79.0 ± 7.3	71.4 ± 12.4	109.2 ± 7.6	45.1 ± 5.0	86.1 ± 7.6
	QC_{MID}	53.9 ± 14.1	79.1 ± 1.9	77.5 ± 13.8	92.7 ± 8.7	41.7 ± 14.5	73.2 ± 6.6
	QC_{HIGH}	51.3 ± 15.7	80.6 ± 6.8	73.7 ± 8.7	96.8 ± 9.6	36.9 ± 9.6	77.5 ± 1.6
**CORT-d_8**	QC_{3xLLOQ}	65.1 ± 9.7	86.5 ± 7.4	91.6 ± 5.6	109.8 ± 2.2	59.4 ± 7.1	95.0 ± 9.4
	QC_{MID}	59.2 ± 7.5	83.2 ± 5.2	86.2 ± 7.3	104.7 ± 8.0	50.7 ± 4.0	86.8 ± 2.3
	QC_{HIGH}	62.8 ± 8.9	90.9 ± 6.7	90.4 ± 8.8	105.7 ± 9.1	56.3 ± 4.3	95.6 ± 3.2
**Cortisol-d_4**	QC_{3xLLOQ}	37.0 ± 2.4	88.0 ± 5.2	111.7 ± 8.1	100.9 ± 7.3	41.2 ± 2.2	88.5 ± 3.4
	QC_{MID}	35.5 ± 5.4	90.4 ± 10.1	113.7 ± 7.4	96.4 ± 10.6	40.3 ± 6.2	86.3 ± 3.0
	QC_{HIGH}	37.1 ± 5.8	92.9 ± 14.9	108.9 ± 12.7	101.5 ± 18.4	40.0 ± 4.0	92.2 ± 4.7
**Cortisone-$^{13}C_3$**	QC_{3xLLOQ}	41.5 ± 1.1	95.5 ± 4.3	102.5 ± 5.9	93.7 ± 6.0	42.5 ± 3.1	89.4 ± 4.8
	QC_{MID}	40.5 ± 6.1	103.0 ± 12.6	104.0 ± 5.6	88.5 ± 9.2	42.0 ± 6.2	90.4 ± 6.2
	QC_{HIGH}	43.0 ± 6.8	107.7 ± 18.0	99.9 ± 12.4	93.7 ± 17.9	42.5 ± 4.4	98.4 ± 4.8
**DHT-d_3**	QC_{3xLLOQ}	82.4 ± 11.3	93.3 ± 8.6	80.0 ± 9.4	107.6 ± 8.4	65.2 ± 4.5	99.9 ± 4.1
	QC_{MID}	73.2 ± 6.3	92.1 ± 10.2	97.0 ± 4.5	112.7 ± 8.7	70.9 ± 5.7	103.2 ± 5.1
	QC_{HIGH}	84.1 ± 12.0	107.6 ± 7.1	77.5 ± 4.8	103.2 ± 9.5	65.0 ± 8.5	110.6 ± 4.4
**E1-$^{13}C_3$**	QC_{3xLLOQ}	90.1 ± 20.0	110.0 ± 14.5	83.4 ± 18.7	100.4 ± 6.2	72.5 ± 8.5	109.9 ± 10.1
	QC_{MID}	97.5 ± 23.3	99.6 ± 8.4	73.4 ± 21.4	102.0 ± 6.0	68.5 ± 17.3	101.4 ± 7.8
	QC_{HIGH}	76.6 ± 19.8	97.7 ± 16.2	81.7 ± 6.9	110.6 ± 18.1	61.8 ± 12.3	105.7 ± 3.2
**E2-$^{13}C_3$**	QC_{3xLLOQ}	74.7 ± 19.3	101.4 ± 3.0	95.7 ± 26.5	95.9 ± 8.3	67.9 ± 7.5	97.2 ± 7.6
	QC_{MID}	56.2 ± 22.0	91.3 ± 7.2	84.3 ± 5.7	98.9 ± 8.0	46.6 ± 14.9	90.2 ± 9.2
	QC_{HIGH}	63.8 ± 14.3	93.2 ± 13.3	92.5 ± 6.2	99.6 ± 12.9	58.8 ± 12.8	91.6 ± 4.7
**E3-d_3**	QC_{3xLLOQ}	75.7 ± 16.6	97.2 ± 13.9	76.6 ± 20.4	92.5 ± 18.3	56.0 ± 9.6	88.6 ± 13.3
	QC_{MID}	55.6 ± 10.6	74.3 ± 13.1	73.3 ± 9.5	93.0 ± 16.5	40.9 ± 10.2	68.6 ± 14.1
	QC_{HIGH}	54.3 ± 0.8	73.8 ± 11.5	82.8 ± 6.6	102.9 ± 18.9	44.9 ± 3.2	74.5 ± 7.0
**Preg-$^{13}C_2$-d_2**	QC_{3xLLOQ}	83.1 ± 27.8	91.0 ± 13.0	89.3 ± 15.5	95.6 ± 9.8	56.4 ± 6.3	86.7 ± 12.6
	QC_{MID}	63.0 ± 17.2	92.1 ± 8.0	96.9 ± 17.2	76.5 ± 8.0	52.1 ± 18.1	70.5 ± 10.3
	QC_{HIGH}	53.9 ± 15.1	85.4 ± 6.1	92.1 ± 10.9	83.2 ± 7.8	46.1 ± 12.0	70.8 ± 4.2
**P-$^{13}C_3$**	QC_{3xLLOQ}	99.9 ± 23.6	111.7 ± 10.2	78.1 ± 21.2	99.4 ± 16.9	75.8 ± 18.7	110.9 ± 20.3

	QC_{MID}	71.1 ± 12.9	91.7 ± 5.2	73.6 ± 8.0	104.6 ± 6.7	51.8 ± 7.3	95.8 ± 6.1
	QC_{HIGH}	73.6 ± 13.4	104.8 ± 7.2	87.9 ± 5.4	110.7 ± 6.7	64.5 ± 11.0	115.8 ± 6.3
	QC_{3xLLOQ}	87.6 ± 15.8	91.2 ± 7.4	89.6 ± 10.6	105.4 ± 10.1	77.2 ± 5.1	95.6 ± 2.3
**17OHP - $^{13}C_3$**	QC_{MID}	81.1 ± 13.3	94.9 ± 4.1	80.5 ± 9.0	97.5 ± 7.6	64.5 ± 6.1	92.4 ± 6.4
	QC_{HIGH}	79.4 ± 16.0	92.0 ± 8.1	84.2 ± 10.4	100.7 ± 10.0	65.5 ± 6.6	92.1 ± 1.3
	QC_{3xLLOQ}	94.6 ± 12.0	100.8 ± 4.8	92.6 ± 9.6	108.8 ± 8.7	87 ± 9.1	109.4 ± 7.4
**T-$^{13}C_3$**	QC_{MID}	74.2 ± 6.7	86.2 ± 9.4	100.1 ± 6.6	108.3 ± 5.1	73.9 ± 4.6	92.9 ± 7.2
	QC_{HIGH}	79.2 ± 14.1	92.1 ± 6.3	92.9 ± 11.7	111.1 ± 11.6	72.3 ± 5.5	101.7 ± 4.0
	QC_{3xLLOQ}	80.6 ± 15.8	91.3 ± 6.9	53.2 ± 7.5	105.6 ± 5.5	42.1 ± 4.0	96.3 ± 6.9
**ChAc**	QC_{MID}	65.4 ± 19.6	96.8 ± 8.9	49.2 ± 7.3	90.5 ± 7.2	31.3 ± 6.7	87.0 ± 1.6
	QC_{HIGH}	62.1 ± 20.8	96.6 ± 13.4	62.7 ± 8.4	102.6 ± 10.5	37.6 ± 10.3	98.0 ± 4.6
	QC_{3xLLOQ}	71.3 ± 12.4	95.0 ± 11.1	107.5 ± 8.0	103.2 ± 4.1	76.1 ± 8.6	97.8 ± 9.5
**Dienogest**	QC_{MID}	65.9 ± 7.3	91.1 ± 7.6	105.7 ± 9.9	99.4 ± 7.8	69.1 ± 3.7	90.1 ± 1.4
	QC_{HIGH}	70.3 ± 10.1	101.7 ± 7.1	110.4 ± 8.5	103.1 ± 6.0	76.9 ± 6.3	104.6 ± 4.0
	QC_{3xLLOQ}	75.9 ± 21.2	112.8 ± 9.8	78.7 ± 25.4	96.6 ± 13.6	55.9 ± 10.6	108.0 ± 8.9
**EE2**	QC_{MID}	80.0 ± 20.3	86.7 ± 5.2	72.1 ± 20.7	98.8 ± 8.9	56.9 ± 21.4	85.3 ± 4.2
	QC_{HIGH}	70.1 ± 21.2	91.1 ± 12.5	92.5 ± 13.1	100.0 ± 12.1	63.9 ± 17.6	89.9 ± 4.6
	QC_{3xLLOQ}	91.8 ± 14.6	98.7 ± 4.0	77.9 ± 9.2	105.1 ± 5.4	70.5 ± 4.6	103.6 ± 2.0
**LNG**	QC_{MID}	78.6 ± 12.9	97.9 ± 5.0	80.1 ± 10.0	102.9 ± 7.3	62.2 ± 6.9	100.5 ± 4.8
	QC_{HIGH}	80.5 ± 14.1	102.7 ± 9.8	80.4 ± 6.3	106.9 ± 10.1	64.1 ± 7.7	109.0 ± 0.9
	QC_{3xLLOQ}	81.5 ± 10.1	100.8 ± 9.2	55.8 ± 6.3	107.6 ± 3.6	45.0 ± 3.1	108.5 ± 12.1
**NoAc**	QC_{MID}	71.2 ± 10.1	107.0 ± 13.4	51.1 ± 6.2	94.2 ± 7.2	36.1 ± 4.9	100.3 ± 9.4
	QC_{HIGH}	71.7 ± 19.9	114.0 ± 9.7	60.7 ± 6.9	99.3 ± 7.2	42.4 ± 8.8	112.9 ± 8.0

[b]Mean and error of 5 different plasma lots measured in triplicate per level are shown, corrected via IS added at same time point of analyte spike.

Table S6. Validation results of precision and accuracy.[c]

Surrogate Calibrant		QC_{LLOQ} Prec. [%]	QC_{LLOQ} Acc. [%]	QC_{3xLLOQ} Prec. [%]	QC_{3xLLOQ} Acc. [%]	QC_{MID} Prec. [%]	QC_{MID} Acc. [%]	QC_{HIGH} Prec. [%]	QC_{HIGH} Acc. [%]
ALLO-d_5	Day 1	4.6	106.9	5.6	105.6	4.4	98.7	7.4	95.9
	Day 2	1.8	107.3	5.8	107.8	7.0	96.1	9.1	104.4
	Day 3	6.4	101.2	3.2	102.5	4.6	95.6	3.1	102.0
	Inter-day	5.0	105.1	5.1	105.3	5.2	96.8	7.5	100.7
CORT-d_8	Day 1	9.9	106.2	10.9	99.8	8.3	91.8	7.9	101.3
	Day 2	7.3	107.3	6.7	110.3	4.1	87.9	2.8	96.6
	Day 3	9.5	103.2	7.5	105.3	6.4	96.1	4.1	103.5
	Inter-day	8.5	105.6	8.7	104.8	7.1	92.0	5.8	100.6
Cortisol-d_4	Day 1	10.1	103.6	4.8	100.5	7.2	97.0	5.6	110.1
	Day 2	7.0	96.8	9.5	102.2	6.7	100.6	3.5	105.7
	Day 3	10.9	102.3	4.8	100.5	7.2	97.0	5.6	110.1
	Inter-day	8.6	102.6	7.0	99.6	7.1	98.6	7.7	104.1
Cortisone-$^{13}C_3$	Day 1	5.8	104.3	5.9	101.1	4.1	102.9	7.3	108.4
	Day 2	4.5	100.2	8.5	106.3	7.3	106.2	7.1	110.0
	Day 3	10.2	106.8	11.6	100.0	2.9	96.1	6.2	96.1
	Inter-day	7.3	103.9	8.7	102.5	6.5	101.7	8.9	104.8
DHT-d_3	Day 1	5.7	108.1	8.8	102.7	7.5	96.6	2.9	109.5
	Day 2	7.4	99.9	8.7	100.7	3.9	98.8	4.5	104.0
	Day 3	5.4	96.8	6.0	95.5	4.6	99.2	3.2	103.5
	Inter-day	7.5	101.6	8.1	99.6	5.2	98.5	4.3	105.7
E1-$^{13}C_3$	Day 1	10.0	102.7	7.7	104.8	6.6	96.3	9.1	98.2
	Day 2	12.1	107.8	8.8	94.4	8.5	94.3	8.0	97.8
	Day 3	7.4	109.7	7.8	98.7	8.1	107.0	5.9	105.6
	Inter-day	11.4	104.9	8.8	99.6	9.3	99.2	8.0	100.5
E2-$^{13}C_3$	Day 1	9.9	95.3	5.0	104.0	4.1	95.3	7.5	97.6
	Day 2	7.5	93.3	7.3	88.9	10.4	96.1	8.6	101.4
	Day 3	6.3	104.0	5.8	97.5	4.0	101.2	8.5	103.3
	Inter-day	9.1	97.8	8.6	96.8	7.0	97.8	8.3	100.5
E3-d_3	Day 1	6.7	109.8	9.2	96.7	7.4	94.0	4.6	91.2
	Day 2	10.4	103.5	8.5	106.5	6.3	90.0	4.1	87.4
	Day 3	8.5	104.6	7.9	103.5	4.6	96.7	5.8	104.1
	Inter-day	8.6	105.8	8.9	102.2	6.4	93.9	9.1	94.3
Preg-$^{13}C_2$-d_2	Day 1	6.4	97.0	3.2	89.2	4.6	104.8	3.1	94.9
	Day 2	1.8	102.9	5.8	93.8	7.0	105.3	9.1	97.1
	Day 3	4.6	102.5	5.6	91.9	4.4	108.2	7.4	89.2
	Inter-day	5.0	105.1	5.1	105.3	5.2	96.8	7.5	100.7
P-$^{13}C_3$	Day 1	5.3	102.0	5.1	100.8	7.6	93.9	2.5	91.0
	Day 2	12.3	98.2	10.9	101.3	5.1	101.7	3.0	107.8
	Day 3	5.3	113.6	9.4	102.5	4.3	95.5	3.8	100.9
	Inter-day	9.9	104.6	8.2	101.6	6.4	97.0	7.7	99.9

17OHP-$^{13}C_3$	Day 1	9.6	97.4	6.6	103.6	3.9	95.4	6.8	100.9
	Day 2	9.3	109.6	4.7	107.1	9.7	96.8	5.6	108.4
	Day 3	7.6	100.7	4.3	106.0	8.9	98.3	8.9	102.7
	Inter-day	9.5	103.5	5.1	106.0	7.4	96.7	7.3	104.0
T-$^{13}C_3$	Day 1	5.9	109.3	3.5	109.3	6.1	95.4	5.3	106.3
	Day 2	7.8	97.1	9.3	99.1	6.7	98.3	4.3	110.1
	Day 3	12.3	102.0	2.7	111.4	5.5	92.3	10.0	99.0
	Inter-day	9.9	103.1	7.4	106.5	7.0	94.3	7.7	105.1
ChAc	Day 1	4.7	114.5	1.9	102.3	5.9	95.7	3.1	98.4
	Day 2	5.0	112.3	4.3	104.7	3.6	96.2	6.7	100.9
	Day 3	7.0	102.7	3.8	90.4	3.7	98.9	4.1	101.3
	Inter-day	7.7	109.2	7.3	99.1	4.4	97.0	4.7	100.2
Dienogest	Day 1	6.5	111.1	9.8	108.1	6.1	94.2	9.6	98.1
	Day 2	4.6	110.4	2.8	103.9	6.7	93.0	2.1	95.4
	Day 3	4.0	100.9	6.1	99.6	3.8	100.2	6.8	101.1
	Inter-day	6.5	107.2	7.3	103.6	6.2	95.9	6.8	98.3
EE2	Day 1	13.5	91.1	6.1	98.4	3.6	93.0	10.1	94.5
	Day 2	9.0	105.8	7.0	97.8	7.5	99.7	8.0	99.1
	Day 3	11.6	102.7	4.5	100.6	7.6	98.4	8.1	103.1
	Inter-day	12.3	99.9	5.6	99.0	6.8	97.1	8.9	98.9
LNG	Day 1	11.6	102.7	9.4	97.0	9.3	89.7	7.4	99.4
	Day 2	9.3	107.4	7.1	100.9	4.8	91.7	3.2	97.5
	Day 3	12.2	99.1	5.3	100.0	6.8	98.7	3.5	103.4
	Inter-day	10.5	104.0	7.1	99.3	7.9	93.4	5.3	100.1
NoAc	Day 1	8.5	89.5	5.2	102.4	5.5	98.6	5.5	87.5
	Day 2	12.1	92.9	6.0	91.6	2.6	90.0	7.0	100.7
	Day 3	10.4	96.7	2.6	96.9	7.6	100.2	5.2	101.0
	Inter-day	11.1	93.7	6.5	97.0	7.2	96.3	8.7	96.4

[c]Non-endogenous compounds (synthetic contraceptives) did not require surrogate calibration and were calibrated via the target analyte.

Table S7. Post-processing stability.[d]

Analyte	QC level	12 h [%]		50 h [%]	
		Peak area	IS ratio	Peak area	IS ratio
	QC_{3xLLOQ}	102.4 ± 7.1	103.6 ± 5.0	82.3 ± 6.5	68.9 ± 6.1
**ALLO-d_5**	QC_{MID}	98.6 ± 3.3	103.8 ± 3.2	92.7 ± 13.5	73.5 ± 14.6
	QC_{HIGH}	116.3 ± 7.3	101.1 ± 5.1	83.9 ± 10.4	100.6 ± 4.9
	QC_{3xLLOQ}	109.6 ± 6.6	108.5 ± 4.7	112.5 ± 8.5	106.5 ± 9.5
**CORT-d_8**	QC_{MID}	99.2 ± 2.8	106.7 ± 4.8	97.5 ± 18.5	94.8 ± 7.0
	QC_{HIGH}	101.7 ± 4.6	103.7 ± 3.1	91.4 ± 11.0	98.3 ± 3.9
	QC_{3xLLOQ}	106.8 ± 11.7	101.4 ± 7.3	92.5 ± 10.6	113.8 ± 7.9
**Cortisol-d_4**	QC_{MID}	100.3 ± 4.1	102.3 ± 9.7	84.9 ± 25.9	115.9 ± 9.8
	QC_{HIGH}	96.9 ± 5.3	97.1 ± 6.1	80.7 ± 13.3	105.8 ± 5.0
	QC_{3xLLOQ}	96.0 ± 9.4	92.0 ± 7.7	97.3 ± 9.9	119.3 ± 5.6
**Cortisone-$^{13}C_3$**	QC_{MID}	103.4 ± 4.2	105.4 ± 10.4	83.2 ± 26.9	118.2 ± 10.2
	QC_{HIGH}	95.6 ± 5.2	95.8 ± 4.7	81.5 ± 14.7	106.9 ± 4.9
	QC_{3xLLOQ}	97.2 ± 8.2	91.8 ± 8.0	62.1 ± 11.9	63.2 ± 17.2
**DHT-d_3**	QC_{MID}	99.0 ± 3.8	101.3 ± 4.6	96.5 ± 7.3	101.1 ± 7.1
	QC_{HIGH}	105.0 ± 3.5	101.7 ± 3.7	92.5 ± 7.6	99.3 ± 3.5
	QC_{3xLLOQ}	81.8 ± 9.9	108.0 ± 7.5	54.0 ± 7.1	115.8 ± 7.6
**E1-$^{13}C_3$**	QC_{MID}	82.6 ± 8.8	106.2 ± 8.9	53.6 ± 29.6	106.8 ± 8.1
	QC_{HIGH}	106.3 ± 12.5	100.2 ± 5.7	75.3 ± 17.2	106.9 ± 7.6
	QC_{3xLLOQ}	72.1 ± 8.0	92.5 ± 6.0	36.2 ± 5.5	78.7 ± 4.2
**E2-$^{13}C_3$**	QC_{MID}	77.3 ± 6.4	99.3 ± 5.5	32.1 ± 5.8	78.2 ± 5.5
	QC_{HIGH}	117.4 ± 7.3	100.9 ± 6.7	66.1 ± 13.3	103.0 ± 9.8
	QC_{3xLLOQ}	111.5 ± 9.1	109.9 ± 5.7	83.9 ± 10.1	80.5 ± 6.2
**E3-d_3**	QC_{MID}	96.8 ± 3.8	104.0 ± 5.5	102.3 ± 9.0	92.3 ± 19.2
	QC_{HIGH}	107.5 ± 3.8	109.6 ± 4.9	78.9 ± 8.6	84.7 ± 4.7
	QC_{3xLLOQ}	93.6 ± 7.0	94.5 ± 8.8	83.9 ± 6.8	70.6 ± 6.7
**Preg-$^{13}C_2$-d_2**	QC_{MID}	84.5 ± 7.9	90.7 ± 8.3	53.0 ± 4.3	41.8 ± 3.1
	QC_{HIGH}	107.9 ± 5.2	93.6 ± 4.5	73.7 ± 10.8	89.0 ± 6.5
**P-$^{13}C_3$**	QC_{3xLLOQ}	105.0 ± 9.9	107.2 ± 12.4	144.2 ± 16.9	90.3 ± 16.8

	QC_{MID}	102.3 ± 3.5	103.6 ± 2.3	139.2 ± 13.7	100.9 ± 5.0
	QC_{HIGH}	115.2 ± 4.6	104.7 ± 2.4	90.7 ± 8.9	106.6 ± 2.0
	QC_{3xLLOQ}	102.7 ± 7.3	106.7 ± 11.2	92.0 ± 11.4	99.5 ± 11.9
**17OHP -$^{13}C_3$**	QC_{MID}	101.0 ± 3.5	100.9 ± 3.6	96.4 ± 14.4	114.2 ± 5.8
	QC_{HIGH}	102.1 ± 4.9	98.3 ± 6.6	91.3 ± 11.7	112.4 ± 5.5
	QC_{3xLLOQ}	102.2 ± 5.1	106.9 ± 5.6	107.0 ± 8.4	114.7 ± 5.8
**T-$^{13}C_3$**	QC_{MID}	98.4 ± 1.6	98.3 ± 4.1	101.0 ± 11.6	118.3 ± 5.0
	QC_{HIGH}	98.9 ± 2.7	95.2 ± 6.0	96.7 ± 7.1	118.6 ± 5.0
	QC_{3xLLOQ}	100.3 ± 3.7	101.4 ± 3.9	124.9 ± 6.9	105.3 ± 3.2
**ChAc**	QC_{MID}	98.3 ± 4.1	103.3 ± 2.1	122.6 ± 1.7	97.8 ± 3.3
	QC_{HIGH}	116.9 ± 5.5	101.5 ± 5.9	84.4 ± 9.4	101.6 ± 5.2
	QC_{3xLLOQ}	107.2 ± 9.2	106.0 ± 4.9	106.2 ± 8.1	100.5 ± 6.9
**Dienogest**	QC_{MID}	99.6 ± 1.8	107.0 ± 4.3	103.6 ± 11.8	98.6 ± 5.9
	QC_{HIGH}	99.4 ± 3.8	101.4 ± 4.1	95.7 ± 7.4	102.4 ± 4.3
	QC_{3xLLOQ}	80.7 ± 6.7	108.6 ± 3.1	30.2 ± 6.9	117.8 ± 5.3
**EE2**	QC_{MID}	76.2 ± 14	97.9 ± 8.8	20.7 ± 10.2	96.9 ± 6.2
	QC_{HIGH}	129.6 ± 3.8	107.7 ± 6.0	61.3 ± 17.9	93.4 ± 6.4
	QC_{3xLLOQ}	117.7 ± 7.5	112.2 ± 6.0	101.5 ± 11.3	103.6 ± 9.9
**LNG**	QC_{MID}	103.3 ± 2.0	105.8 ± 6.2	101.1 ± 9.6	106.8 ± 5.8
	QC_{HIGH}	103.8 ± 3.7	100.6 ± 3.5	91.1 ± 7.7	97.8 ± 3.6
	QC_{3xLLOQ}	100.8 ± 4.7	101.9 ± 3.4	95.9 ± 5.6	80.5 ± 4.9
**NoAc**	QC_{MID}	99.7 ± 3.4	104.8 ± 2.4	98 ± 4.9	78.3 ± 4.3
	QC_{HIGH}	111.9 ± 3.2	97.1 ± 5.9	83 ± 9.7	100.2 ± 6.7

[d]4 determinations per level, storage in 4 °C autosampler. Degradation of DMIS products. IS correction not efficient after 50 h if IS is not labelled analogue of target analyte.

Table S8. Steroid profiles and reference intervals for endogenous hormones.

**Steroids (ng/mL)**	**NC**, median (IQR)	**IUD**, median (IQR)	**OC**, median (IQR)	**Reference Intervals**[e]
E1	1.00 (0.95)	0.60 (0.64)	0.14 (0.11)	0.02 – 0.25[1]
17OHP	0.88 (0.93)	0.36 (0.68)	0.09 (0.11)	0.15 – 2.9[1]
Cortisol	69.38 (36.05)	51.59 (21.31)	110.78 (51.83)	35.16 – 460.69[2]
P	6.10 (9.15)	2.64 (3.02)	0.15 (1.69)	≤ 31.4[1]
ALLO	0.37 (0.47)	0.09 (0.42)	0.07 (0.09)	0.04 – 1.18[3]
Cortisone	13.13 (7.11)	11.38 (4.96)	19.24 (13.13)	10.09 – 39.29[2]
Preg	5.65 (5.18)	2.64 (3.02)	1.45 (2.05)	0.10 – 9.39[2]
DHT	0.11 (0.14)	0.07 (0.06)	0.07 (0.12)	0.05 – 0.46[1]
E2	0.11 (0.07)	0.07 (0.09)	0.03 (0.06)	0.02 – 0.75[1]
T	0.15 (0.19)	0.12 (0.12)	0.13 (0.11)	0.02 – 0.45[1]
CORT	1.90 (2.59)	1.90 (1.57)	1.88 (3.76)	0.42 – 50.79[2]
E3	0.00 (0.00)	0.00 (0.00)	0.00 (0.00)	NA[f,1]
EE	-	-	0.09 (0.07)	-
ChAc	-	-	0.49 (0.44)	-
Dienogest	-	-	16.10 (4.43)	-
LNG	-	0.06 (0.04)	2.39 (1.83)	-

[e] values from adult females, excluding pregnant and post-menopausal women

[f] No values for non-pregnant females

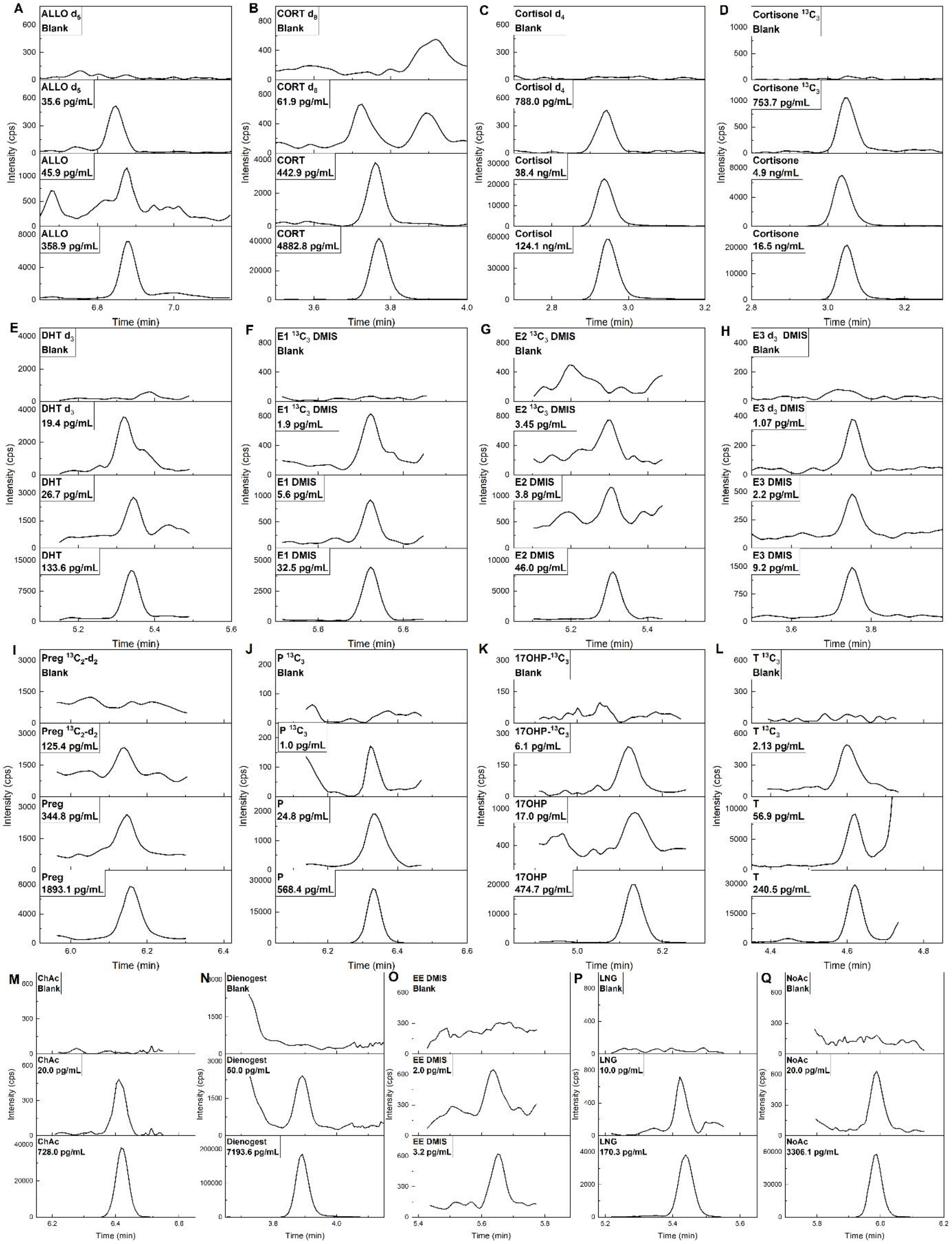
A
ALLO d5 Blank
ALLO d5 35.6 pg/mL
ALLO 45.9 pg/mL
ALLO 358.9 pg/mL
B
CORT d8 Blank
CORT d8 61.9 pg/mL
CORT 442.9 pg/mL
CORT 4882.8 pg/mL
C
Cortisol d4 Blank
Cortisol d4 788.0 pg/mL
Cortisol 38.4 ng/mL
Cortisol 124.1 ng/mL
D
Cortisone 13C3 Blank
Cortisone 13C3 753.7 pg/mL
Cortisone 4.9 ng/mL
Cortisone 16.5 ng/mL
E
DHT d3 Blank
DHT d3 19.4 pg/mL
DHT 26.7 pg/mL
DHT 133.6 pg/mL
F
E1 13C3 DMIS Blank
E1 13C3 DMIS 1.9 pg/mL
E1 DMIS 5.6 pg/mL
E1 DMIS 32.5 pg/mL
G
E2 13C3 DMIS Blank
E2 13C3 DMIS 3.45 pg/mL
E2 DMIS 3.8 pg/mL
E2 DMIS 46.0 pg/mL
H
E3 d3 DMIS Blank
E3 d3 DMIS 1.07 pg/mL
E3 DMIS 2.2 pg/mL
E3 DMIS 9.2 pg/mL
I
Preg 13C2-d2 Blank
Preg 13C2-d2 125.4 pg/mL
Preg 344.8 pg/mL
Preg 1893.1 pg/mL
J
P 13C3 Blank
P 13C3 1.0 pg/mL
P 24.8 pg/mL
P 568.4 pg/mL
K
17OHP-13C3 Blank
17OHP-13C3 6.1 pg/mL
17OHP 17.0 pg/mL
17OHP 474.7 pg/mL
L
T 13C3 Blank
T 13C3 2.13 pg/mL
T 56.9 pg/mL
T 240.5 pg/mL
M
ChAc Blank
ChAc 20.0 pg/mL
ChAc 728.0 pg/mL
N
Dienogest Blank
Dienogest 50.0 pg/mL
Dienogest 7193.6 pg/mL
O
EE DMIS Blank
EE DMIS 2.0 pg/mL
EE DMIS 3.2 pg/mL
P
LNG Blank
LNG 10.0 pg/mL
LNG 170.3 pg/mL
Q
NoAc Blank
NoAc 20.0 pg/mL
NoAc 3306.1 pg/mL
Intensity (cps)
Time (min)

Fig. S1. Extracted ion chromatograms of steroids. For endogenous hormones: EICs (top to bottom) show surrogate calibrants in a blank matrix, matrix spiked at LLOQ levels, and targets in real samples quantified at both low and high concentrations. For synthetic hormones, EICs (top to bottom) of targets in blank matrix, matrix spiked at LLOQ levels, and in real samples are shown. A: ALLO; B: CORT; C: cortisol; D: cortisone; E: DHT; F: E1 DMIS; G: E2 DMIS; H: E3 DMIS; I: Preg; J: P; K: 17OHP; L: T; M: ChAc; N: dienogest; O: EE DMIS; P: LNG; Q: NoAc. Visual interpretation at the LLOQ may be affected by differences in background and selectivity between surrogates calibrants and target analytes. Manual review and integration were applied to minimize potential interference, particularly at the low end of the calibration range.

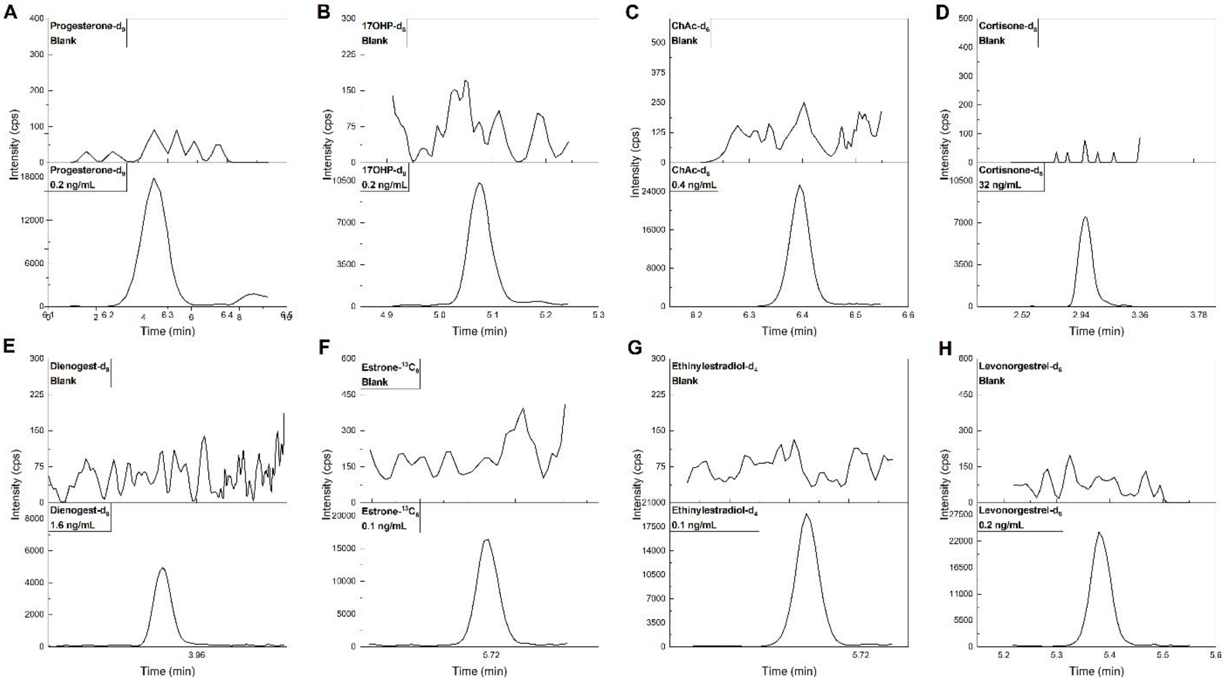

Fig. S2. Extracted ion chromatograms of internal standards in blank and spiked matrix. A: P-d_9; B: 17OHP-d_8; C: ChAc-d_6; D: cortisone-d_8; E: Dienogest-d_8; F: E1-$^{13}C_6$; G: EE-d_4; H: LNG-d_6

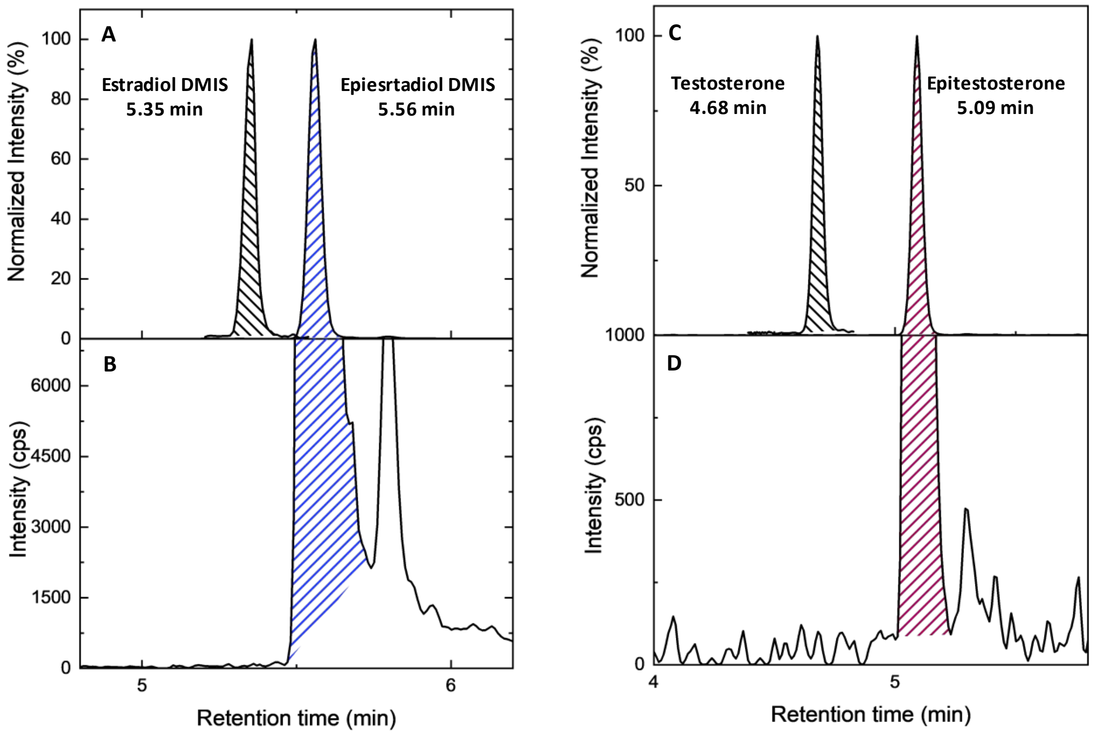

Fig. S3. Chromatographic selectivity for steroid epimers. The injection of E2-DMIS and epiE2-DMIS (A) and T and epiT (C) demonstrated baseline separation of peaks for both pairs of epimers. Injection of only epiE2-DMIS (B) or only epiT (D) confirmed no interference with target analytes.

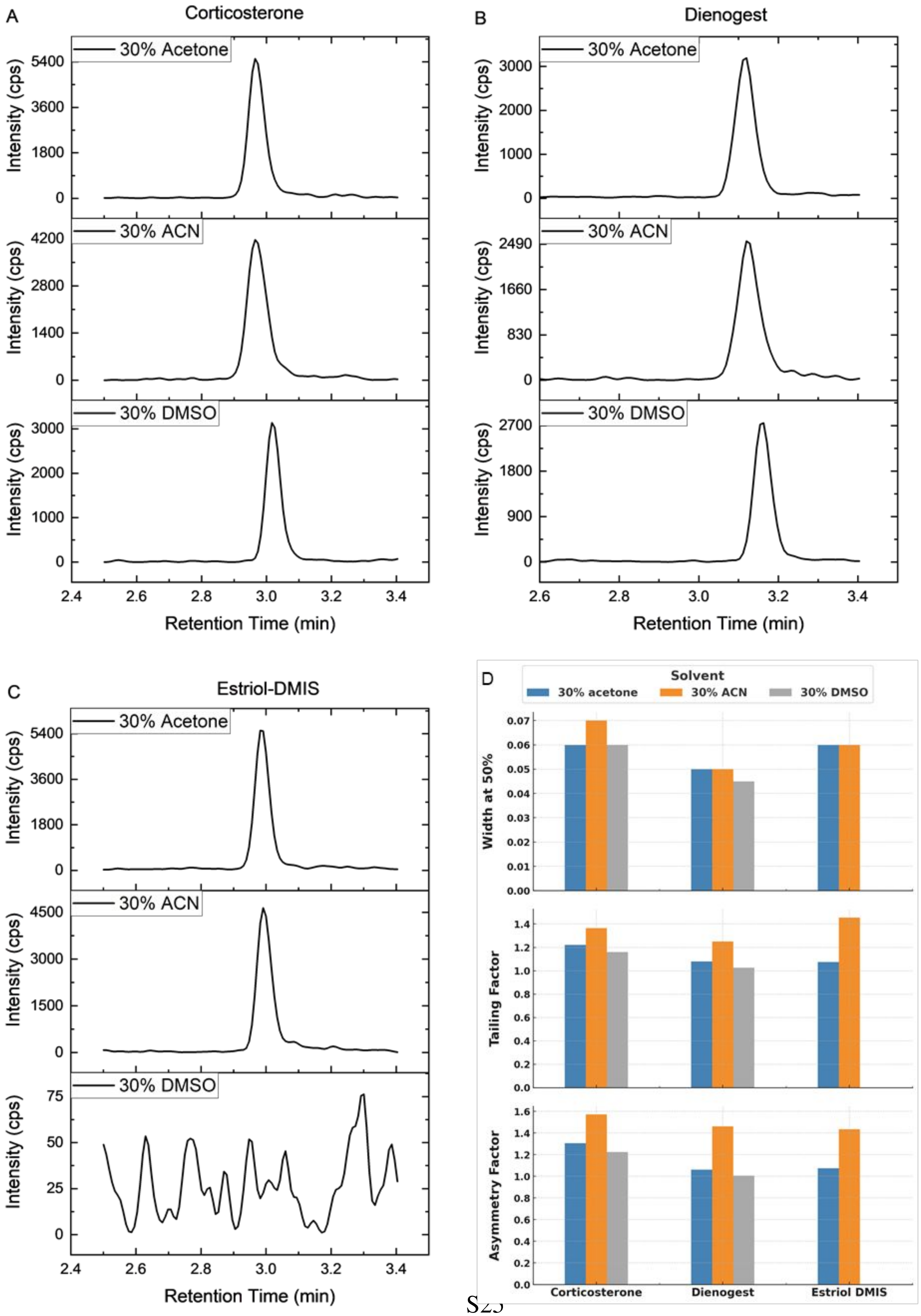
A
Corticosterone
30% Acetone
30% ACN
30% DMSO
Intensity (cps)
Retention Time (min)
B
Dienogest
30% Acetone
30% ACN
30% DMSO
Intensity (cps)
Retention Time (min)
C
Estriol-DMIS
30% Acetone
30% ACN
30% DMSO
Intensity (cps)
Retention Time (min)
D
Solvent
30% acetone
30% ACN
30% DMSO
Width at 50%
Tailing Factor
Asymmetry Factor
Corticosterone
Dienogest
Estriol DMIS

Fig. S4. Optimization of derivatization solvents. Extracted ion chromatograms (EICs) of A) corticosterone (*m/z* 374.3/121.1), B) dienogest (*m/z* 312.2/161.1), and C) estriol DMIS (*m/z* 447.1/383.0) are shown for different derivatization solvents: 30% acetone, 30% ACN, and 30% DMSO. D) shows the comparison of peak shape metrics (n=3): peak width at 50%, tailing factor, and asymmetry factor for each target across the three solvents. Acetone and DMSO offered better peak shapes for early-eluting compounds (corticosterone and dienogest). However, DMSO suffered from insufficient reaction efficiency, as no peak for estriol-DMIS was observed.

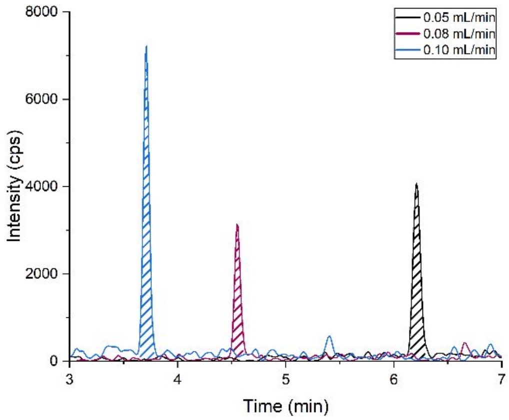

Fig. S5. Flow optimization. Extracted chromatograms of E3-DMIS at flow rates of 0.05, 0.08, and 0.10 mL/min.

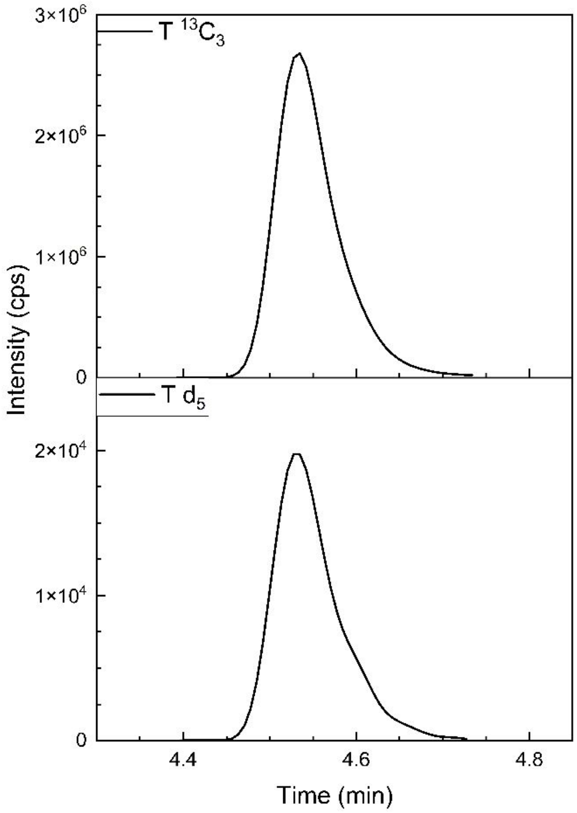

Fig. S6. Isotopic interference between T-d_5 and the M+2 peak of T-$^{13}C_3$. Extracted ion chromatograms (EICs) of T-$^{13}C_3$ (top) and T-d_5 (bottom) from T-$^{13}C_3$ standard solution at Cal 7 concentration.

Reference

(1) Rifai, N. 6th ed NV. Elsevier Health Sciences: Saintt Louis 2017.

(2) Eisenhofer, G.; Peitzsch, M.; Kaden, D.; Langton, K.; Pamporaki, C.; Masjkur, J.; Tsatsaronis, G.; Mangelis, A.; Williams, T. A.; Reincke, M.; Lenders, J. W. M.; Bornstein, S. R. *Clin. Chim. Acta* **2017**, *470* (June 2016), 115–124.

(3) Nyberg, S.; Bäckström, T.; Zingmark, E.; Purdy, R. H.; Poromaa, I. S. *Gynecol. Endocrinol.* **2007**, *23* (5), 257–266.
